# Vanadium-Based Materials in Metal–Ion Hybrid Batteries—Beyond Conventional Lithium-Ion Storage: A Review and Perspectives

**DOI:** 10.1021/acs.energyfuels.5c06570

**Published:** 2026-02-18

**Authors:** Daniela Söllinger, Julie Lam Chen, Ivana Zlatic, Simone Pokrant, Daniel Rettenwander

**Affiliations:** † Department of Materials Science and Engineering, 8018 Norwegian University of Science and Technology, 7034 Trondheim, Norway; ‡ Chemistry and Physics of Materials, 27257 University of Salzburg, 5020 Salzburg, Austria

## Abstract

Metal–ion hybrid batteries represent an emerging class of electrochemical energy-storage devices that combine various charge carriers within a single cell while maintaining electrochemical performance competitive with commercial batteries. Achieving stable and high-performance behavior in hybrid batteries such as Li–Na, Li–Mg, Na–Mg, and Na–Zn requires electrode materials capable of reversible inter- and deintercalation of various ion species. In this context, vanadium-based materials are particularly promising due to their multiple oxidation states, extensive compositional flexibility that enables the intercalation of various ions, and synthetic versatility that allows modifications like doping, ion preintercalation, or composite formation. This perspective provides a comprehensive assessment of the structural and electrochemical properties of vanadium-based materials in metal–ion hybrid batteries. It analyzes the complexities of mixed-ion intercalation mechanisms and identifies key parameters that govern the intercalation behavior in this material class. These insights offer a valuable framework for advancing vanadium-based and other transition-metal electrode materials in hybrid batteries.

## Graphical abstract

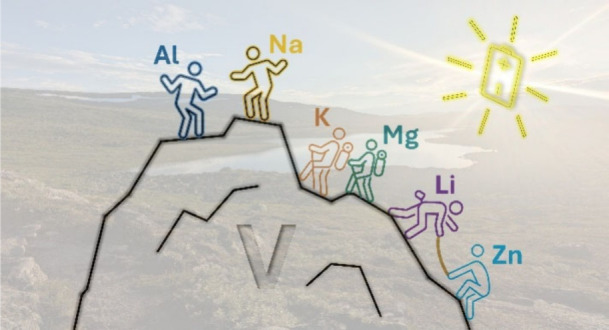

## 1 Introduction

Batteries play a crucial role in modern society enabling the efficient storage of energy from renewable sources such as wind and hydropower and delivering it on demand.^1–3^ Among the various types, lithium-ion batteries (LIBs) are the most widely used, since lithium (Li) has a high gravimetric specific capacity (3861 mAh·g^–1^), low redox potential (−3.04 V vs the standard hydrogen electrode, SHE) and Li-ions have a small ionic radius (0.76 Å).^4–6^ However, lithium is considered a critical material due to its limited global availability—being mainly concentrated in Chile, Australia, Argentina, and China—and the environmentally harmful methods required for its extraction.^7–10^ This geographic concentration raises concerns about resource security, strategic trade dependencies, and supply disruptions, thereby driving initiatives to develop alternative battery types.^11^ These alternative battery types need to maintain a comparable electrochemical performance to LIBs while improving resource accessibility and sustainability.^12,13^ Potential alternative charge carriers include Na, K, Mg, Al, and Zn (Table 1).^4,13–19^ Although these elements are more abundant than Li, they generally have higher redox potentials, and some of them face significant challenges when used in an electrochemical cell.

**Table 1:** Comparison of Several Metals That Are Currently Explored as Electrode Materials in Research^4,14–19^

	Potential vs SHE [V]	Gravimetric specific capacity [mAh·g^–1^]	Volumetric specific capacity [mAh·cm^–3^]	Ionic radii [Å]	Abundancy [10^–4^ wt %]	Charge
Li	–3.04	3861	2066	0.76	22	1+
K	–2.93	685	586	1.38	28,650	1+
Na	–2.71	1165	1127	1.02	25,670	1+
Mg	–2.37	2205	3833	0.72	13,510	2+
Al	–1.66	2980	8063	0.54	77,440	3+
Zn	–0.76	820	5851	0.74	52	2+

Na, and K ions are monovalent like Li ions, but their larger ionic radius often leads to higher stresses on the electrode materials during inter- and deintercalation, resulting in an increasing structural instability and hence fast performance loss.^20,21^ However, the incorporation of a controlled amount of these ion species can be beneficial as their larger ionic radii promote the expansion of the crystal structure. This structural expansion enlarges the intercalation channels, thereby enhancing the intercalation of smaller ions, such as Li ions.

Multivalent ions, such as Mg, Zn, or Al ions, offer higher volumetric capacities than Li ions, but accomplishing battery types with those metals is often more challenging due to the strong electrostatic interactions with the host materials (especially, e.g., oxides). For instance, Mg ion insertion and diffusion in electrode materials are often more hindered than Li ions due to the polarization effects generated by the divalent charge.^22,23^

In addition, magnesium-ion batteries (MIBs) face fundamental challenges that arise primarily from the incompatibility between metallic magnesium and conventional electrolytes, which often leads to the formation of a passivation layer that blocks ion transport. Overcoming this requires complex electrolyte formulations that can stabilize Mg metal, while allowing reversible Mg-ion plating. Furthermore, cathode materials must maintain structural and chemical stability under these harsh electrochemical conditions and enable the reversible insertion and extraction of divalent ions, which is an inherently difficult process due to the strong Coulombic interactions between Mg ions and the host lattice. As a result, multivalent ion batteries often exhibit sluggish ion diffusion, high overpotentials, and poor cycling stability.

Despite these limitations, multivalent ions such as Mg, Zn, or Al ions are highly attractive because their multiple charge carriers offer, in principle, theoretical volumetric capacities up to four times higher than those of Li ions, combined with the advantage of material abundance and safety. To fully exploit these benefits, it is essential to mitigate the intrinsic kinetic and thermodynamic limitations of pure multivalent systems.

To overcome these barriers, hybrid or dual-ion battery batteries have emerged as a promising approach that combines the advantages of multiple ion species within a single electrochemical cell.^18,24^ By integrating both monovalent (e.g., Li, Na, and K ions) and multivalent ions (e.g., Mg, Zn, and Al ions), hybrid batteries can balance the fast kinetics of monovalent ion intercalation with the high-capacity potential of multivalent carriers. This synergistic interaction alleviates several critical issues: it reduces structural strain associated with large ion insertion, stabilizes electrode interfaces, and enables the use of simpler electrolytes that would otherwise be incompatible with pure multivalent battery types.

Thus, hybrid batteries bridge the performance gap between conventional Li-ion and next-generation multivalent battery types, offering a pathway to high energy density, improved cycling stability, and enhanced material sustainability. The rational design of such batteries, including the choice of electrode materials (e.g., vanadium-based materials), compatible electrolytes, and optimized ion-pair chemistries, represent a pivotal step toward realizing cost-effective and resource-resilient energy storage technologies.

## 2 Working Principle of Hybrid Batteries

In hybrid batteries, the electrodes and electrolytes are chosen in such a way that two different types of charge carriers take part in the redox processes. The selected electrolyte(s) or the assigned electrodes can, in principle, enclose various ions (see Figure 1). The commercialized battery setup (Figure 1a) consists of a liquid electrolyte containing a dissolved salt of the intercalating ion and two electrodes capable of reversible inter- and deintercalation.

**Figure 1:**
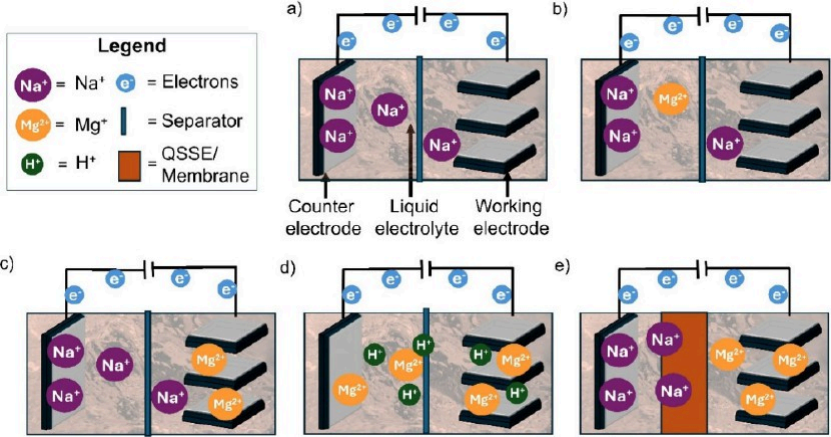
Ion distribution (in electrodes and/or electrolyte(s)) based on the type of hybrid battery. (a) Configuration of a traditional single-ion type battery. (b) A second ion type was added and dissolved in the electrolyte. (c) A second ion type was introduced in (at least) one of the electrodes. (d) Cointercalation of multivalent ions and protons. (e) A quasi-solid state electrolyte (QSSE)/membrane was combined with a liquid electrolyte, which permits the passage of one specific ion type.

In the case of hybrid batteries (Figure 1b and c), a second type of metal ion, either introduced through one of the electrodes or dissolved in the electrolyte, is incorporated into the electrochemical cell.^25,26^ This approach allows the combination of multiple metal ions within a single battery cell. Consequently, lithium can be partially substituted while maintaining an electrochemical performance comparable to that of conventional LIBs. Another type of hybrid battery involves the cointercalation of protons (H^+^) within the electrode (Figure 1d). This type is already used in multivalent ions (such as Mg ions^27,28^ ) or other aqueous-based batteries (e.g., iron–ion batteries^29^) where the cointercalation of H^+^/water improves ionic mobility and the overall electrochemical performance.

A further variation integrates a liquid electrolyte with a quasi-solid-state electrolyte (QSSE) or membrane (Figure 1e), enabling selective ion transport and enhanced safety.^30^ The first metal–ion hybrid battery (similar to Figure 1c) was established by Barker et al. in 2006.^31^ This electrochemical cell employed 1 M LiPF_6_ in ethylene/dimethyl carbonate (EC/DMC) as the electrolyte, graphite as the counter electrode (anode), and Na_3_V_2_(PO_4_)_2_F_3_ (NVPF) as the working electrode (cathode). During the galvanostatic cycling, Na ions were supplied by the NVPF cathode, while Li ions originated from the electrolyte. This configuration led to a specific capacity of 115–120 mAh·g^–1^ over 100 cycles at 0.5C, which is comparable to LIBs today, demonstrating the potential of combining a Li-based anode with a Na-based cathode, which is normally electrochemically incompatible. Barker et al. reported that, despite the limited understanding of the cathode insertion processes, a Li/Na cointercalation mechanism is likely involved.

Indeed, metal–ion hybrid batteries are generally categorized into three main types: Daniel, cointercalation, and rocking chair types (Figure 2). Each type consists of a working electrode (M), a counter electrode (N), and an electrolyte containing two types of metal ions (e.g., Mg and Na ions).

- Daniel type (see Figure 2a): Each electrode interacts exclusively with one type of ion. For example, Mg ions intercalate into electrode M, while Na ions intercalate into electrode N.
- Cointercalation type (see Figure 2b): The working electrode M accommodates the reversible intercalation of various ion species (e.g., Mg and Na ions), whereas the counter electrode N remains selective for a single ion type (e.g., Na ions).
- Rocking chair type (see Figure 2c): Both electrodes facilitate the reversible inter- and deintercalation of various ion types, thereby enabling efficient ion transport between the electrodes during the electrochemical process.

**Figure 2:**
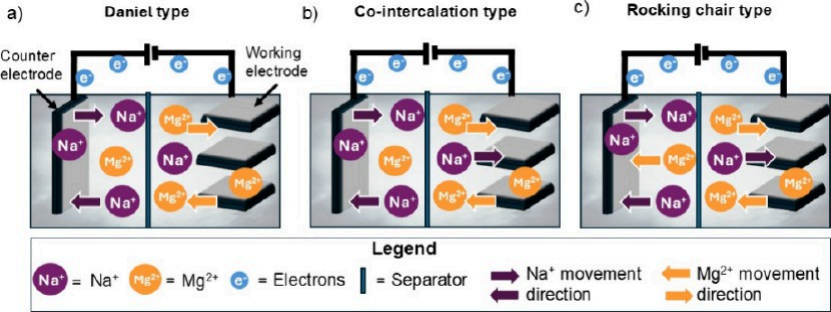
Illustration of the three metal–ion hybrid battery types. Each cell consists of a working electrode (M), a counter electrode (N), and an electrolyte containing two metal ions (Na and Mg ions). (a) Daniel type: each electrode hosts only one ion type. (b) Cointercalation type: M accommodates various ions (Mg and Na ions), while N is selective for one type (Na ions). (c) Rocking chair type: both electrodes reversibly intercalate various ions, enabling bidirectional ion transport.^18^

To achieve stable and promising electrochemical properties in any of these hybrid battery types, electrode materials must support the reversible intercalation of various ions. Transition metals such as titanium (Ti),^32–35^ vanadium­(V),^36–38^ manganese (Mn),^39,40^ iron (Fe),^41,42^ and molybdenum (Mo)^43,44^ fulfill these requirements and are already employed in various hybrid batteries.

Although several reviews of metal–ion hybrid batteries exist, they typically provide broad overviews without in-depth discussion of individual components.^18,24,25,45^ For vanadium-based materials, this is of particular interest due to their multiple accessible oxidation states (+II to + V)^46^ and the wide structural and compositional diversity. They are utilized in a number of battery systems, including commercial single-ion batteries,^47,48^ redox-flow batteries,^49^ and hybrid batteries. Furthermore, many vanadium-based materials allow reversible migration of various ions (e.g., Li , Na , Zn, and Al ions)^47,48,50^ making them promising for the investigation and optimization in hybrid batteries. However, with respect to the numerous achievements of vanadium-based materials as electrode materials in batteries, it should also be mentioned that vanadium has been listed as a critical material in Europe since 2017.^7,51,52^ The reason why elements are listed as critical materials is mainly caused by their risk of supply shortage and their impact on the economy. The limited number of suppliers affects also the criticality of other elements such as magnesium, although it shows much higher abundancies in the Earth’s crust compared to other elements (see Table 1). Therefore, magnesium has been listed as a critical material for already over 10 years. This shows that the abundancy and accessibility to the elements alone are not the crucial factor. In the case of vanadium, the production of vanadium is concentrated in a few countries such as China, Russia, or South Africa.^48^ Concerning the abundancy in the Earth’s crust, vanadium has a higher abundancy than elements such as lithium, cobalt, or nickel.^14,48,53^ In addition, the accessibility is less limited, since vanadium resources are available in minerals (such as basic/intermediate rocks) worldwide, which supports the idea that vanadium-based materials could become highly investigated in the field of batteries. Nevertheless, production facilities must be established in other countries to ensure long-term security of vanadium supply chains and to prevent excessive cost increases. Furthermore, Rogers et al. described that a ‘living’ database, sharing vanadium production and processing routes, could help identify untapped reserves available for extraction and thereby improve the current supply chain of vanadium.^53^

In this perspective, we provide a comprehensive overview of vanadium-based materials in metal–ion hybrid batteries. Beyond the widely studied Li-, Na-, and Mg-ion hybrid batteries, we further highlight the roles of other ions such as Zn, K, and Al ions. In addition, we show the potential of hybrid batteries in combination with membranes/QSSEs as this material class results in inherent safety, as opposed to standard commercial liquid electrolytes. We provide a comprehensive overview of the structural and electrochemical properties of these materials and critically analyze the complexity of the intercalation mechanisms. Finally, we highlight the key factors that are responsible for the intercalation behavior in vanadium-based materials in various metal–ion hybrid batteries.

## 3 Vanadium-Based Materials

The multiple accessible oxidation states of vanadium (+II to + V),^46^ its high theoretical specific capacities in a variety of battery types, e.g., V_2_O_5_ (∼300 mAh·g^–1^ in LIBs)^54^ or V_3_O_7_·H_2_O (∼400 mAh·g^–1^ in aqueous zinc-ion batteries (AZIBs)),^55,56^ which surpass those of conventional cathode materials such as LiCoO_2_ ^57^ or LiFePO_4_ ^58^ (150–180 mAh·g^–1^ in LIBs), and its wide structural diversity are the reasons why vanadium-based materials are widely applied in different energy storage systems (i.e., batteries). The abundance of vanadium is comparable to zinc and is higher than that of other elements often used in battery materials, such as Co or Ni.^59^ Vanadium-based materials such as V_2_O_5_ (V^+V^), compounds with mixed oxidation states of V^+V/IV^ such as V_6_O_13_ and V_3_O_7_, and sodium super ionic conductor (NASICON)-type materials like Na_3_V_2_(PO_4_)_3_ are generally stable under ambient conditions.^60,61^ Other vanadium-based materials with lower V oxidation states, such as VO_2_, VS_4_ (V^+IV^), or V_2_O_3_ (V^+III^), show a lower stability under ambient conditions, due to an increased probability to surface oxidation.^62,63^ Therefore, these compounds should be stored in an inert atmosphere (e.g., in a glovebox). Concerning the sustainability aspects of vanadium-based materials, Samarin et al. presented possible recycling methods for Na_3_V_2_(PO_4_)_3_ and modifications in sodium-ion batteries (SIBs), and Tseng et al. demonstrated recently that recovered V_2_O_5_ in AZIBs shows a promising rate capability and cycling stability.^59,64^ Furthermore, the authors forecast that the proposed recovery and reuse strategy could have broad applicability, beyond batteries, to other vanadium-based technologies. In regard to these properties, vanadium-based materials have attracted increasing attention as a functional material in energy-storage devices, which is reflected in the steadily rising number of publications in the past years (see Figure 3a). This increase is (mostly) attributed to the research on vanadium redox-flow batteries and the investigation of vanadium-based materials in (post)­lithium-ion batteries. Figure 3b further illustrates the variety of vanadium-based materials with different chemistries that have already been explored in various battery types.^65^

**Figure 3:**
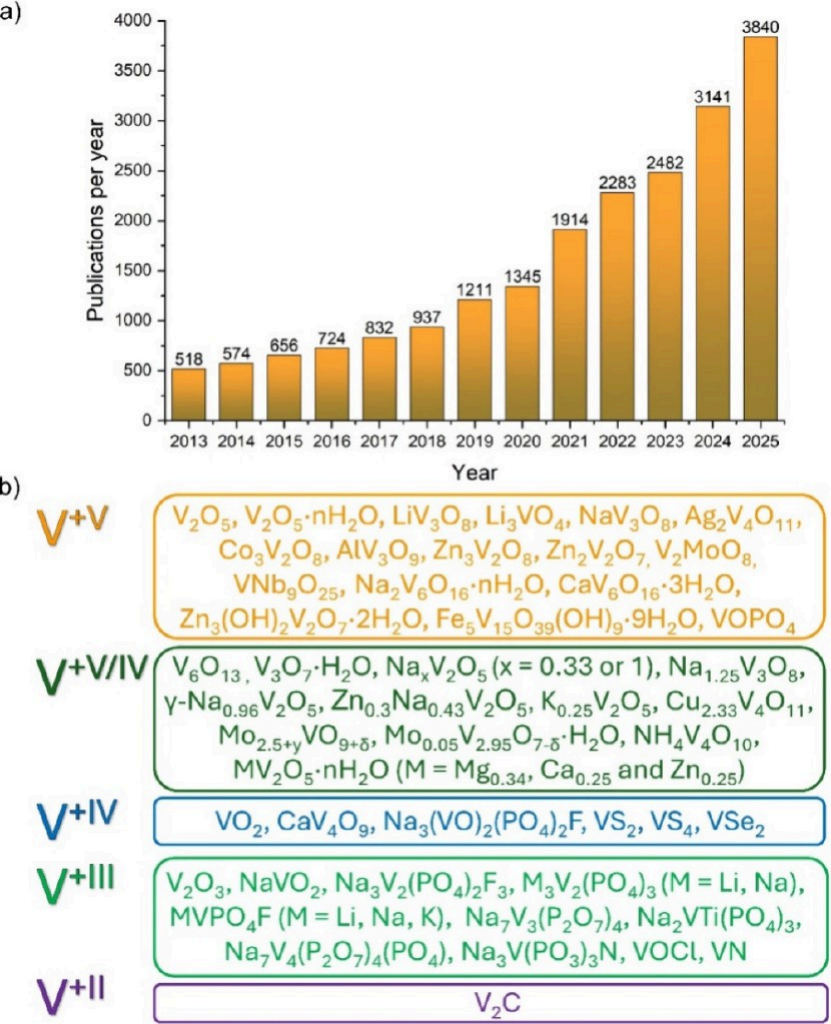
(a) Timeline of the number of papers published worldwide by searching the keywords “vanadium” and “battery” (obtained from Scopus). (b) Vanadium-based materials ordered in regard to their oxidation state.^65^

Many of these materials can be further modified, for example through composite formation by reduced Graphene Oxide (rGO)/mesocarbon microbeads (MCMB)^66^ or alternative synthesis routes that can result in a variation of crystal structures within the same material.^67^ Furthermore, some of the listed vanadium-based materials in Figure 3b can intercalate various ions inside their structure and have already been used and analyzed in hybrid batteries. The crystal structure of several of these vanadium-based materials – namely VO_2_(B) (Crystallography Open Database (COD): 1530870),^68^ V_2_O_5_ (Inorganic Crystal Structure Database (ICSD): 60767),^69^ V_3_O_7_·H_2_O (COD: 2109007),^70^ Na_3_V_2_(PO_4_)_3_ (COD: 2225132),^71^ VS_2_ (ICSD: 651361)^72^ and VS_4_ (COD: 9017794)^73^ – are illustrated in Figure 4.

**Figure 4:**
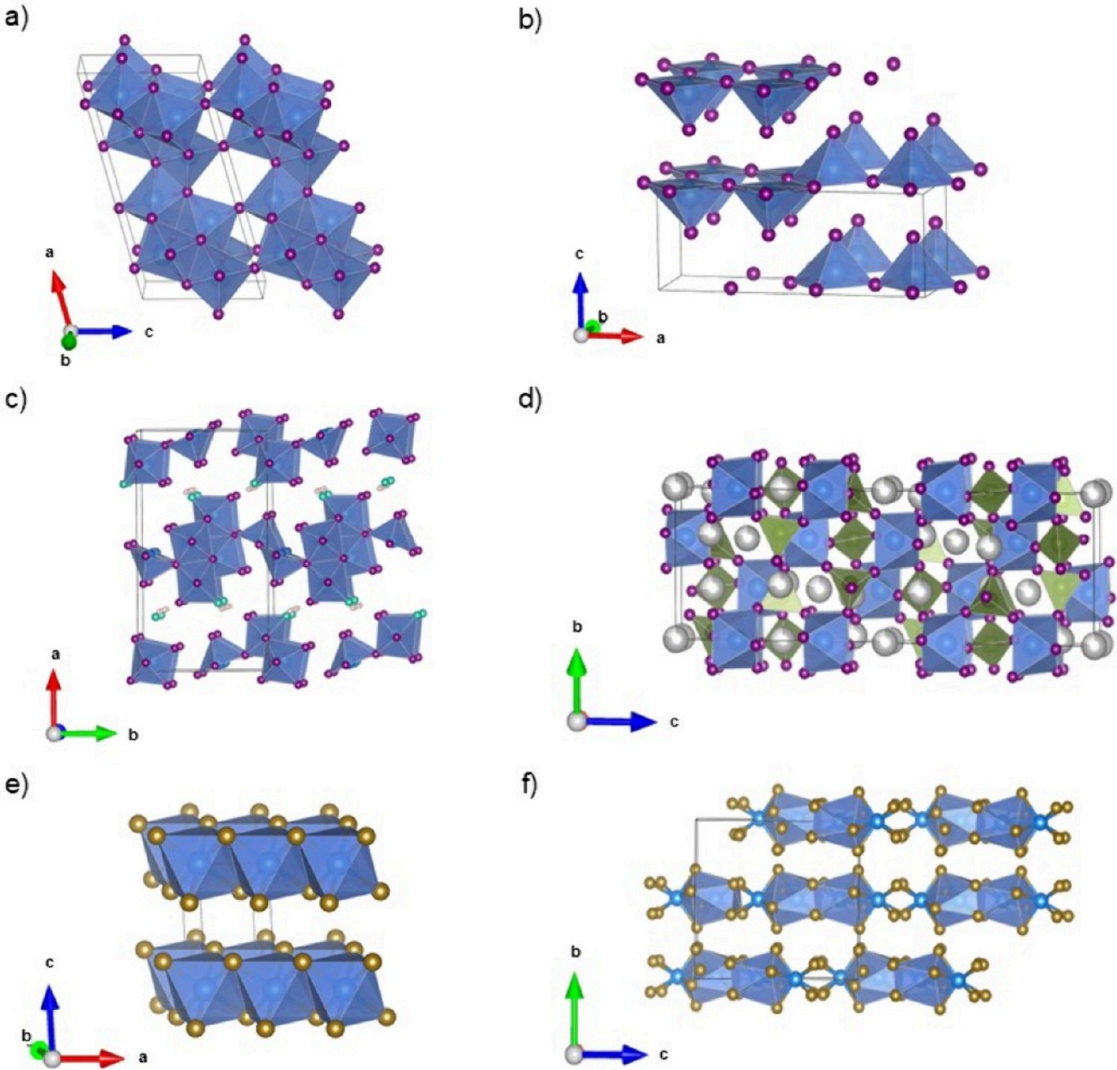
Crystal structures of (a) VO_2_(B) space group *C*2/*m*, (b) V_2_O_5_ space group *PmmnZ*, (c) V_3_O_7_·H_2_O space group *Pnam*, (d) Na_3_V_2_(PO_4_)_3_ space group *R* 3 *c*, (e) VS_2_ space group *P* 3 *m*1, and (f) VS_4_ space group I2/c. Black lines denote one unit cell. V atoms are blue. S atoms are dark yellow. Na atoms are silver. P atoms are olive green. O atoms belonging to H_2_O bonds are turquois. Alll other O atoms are purple. The 3D crystal structures were visualized using VESTA.^80^

Vanadium dioxide (VO_2_) exhibits several polymorphs, including VO_2_(A), VO_2_(B), VO_2_(R), and VO_2_(M), which can be synthesized through various routes such as solid-state or hydrothermal methods.^74^ Among these, VO_2_(B) (*C2/m*, Figure 4a) is most widely used in batteries, especially due to its layered structure and thermal stability.

Vanadium pentoxide (V_2_O_5_; *PmmnZ*, Figure 4b) has a layered structure that facilitates reversible ion intercalation. It is commercially available and can further be modified by introducing structural water or forming nanoparticles, both of which enhance electrochemical performance by improving ion/electron transport and stability.^75,76^

Hydrated vanadium oxide (V_3_O_7_·H_2_O; *Pnam*, Figure 4c) has a layered orthorhombic structure and can be synthesized via a hydrothermal process.^77,78^ In contrast to other vanadium oxides such as VO_2_ and V_2_O_5_, the presence of interlayer hydrogen bonds within the structure increases structural flexibility and stability during the electrochemical measurements.

Sodium vanadium phosphate Na_3_V_2_(PO_4_)_3_ (NVP, *R* 3 *c*, Figure 4d), a NASICON-type material, composes of a three-dimensional network of corner-sharing VO_6_ octahedra and PO_4_ tetrahedra.^71^ It serves as a common cathode material in sodium-ion and hybrid batteries due to its built-in sodium content.

Vanadium disulfide (VS_2_; *P* 3 *m*1, Figure 4e) is usually synthesized via solvothermal or hydrothermal processes.^79^ It offers higher electrical conductivity than other vanadium oxides, enhancing its electrochemical stability.

Lastly, vanadium tetra-sulfide (VS_4_; *I*2*/c*, Figure 4f) has a quasi-one-dimensional (1D) chain-like structure, which is composed of V^(IV+)^ ions coordinated to sulfur dimers.^81^ It is often used in composite electrode materials due to its favorable structural and electrochemical properties.

Composite formation results in significant improvement of the specific capacity and electrochemical stability not only in the case of VS_4_ but also for further vanadium-based materials. Carbon nanotubes (CNT) or reduced graphene oxide (rGO) are used to form composites to improve the electrochemical properties of vanadium-based materials since they often suffer from low electrical conductivity.^82^ Moreover, further modifications including chemical preintercalation of metal ions inside the structure^83,84^ or substituting/doping with (transition) metals^85,86^ have further improved energy densities, specific capacities, and electrochemical stabilities. These enhancements have been demonstrated across various battery types in organic,^87^ aqueous,^88^ and solid-state electrolytes,^89^ with some vanadium-based materials exhibiting potential for use as both cathode and anode.^90,91^

This summarizes how essential vanadium-based materials are in batteries and the possibilities they offer. In fact, the broad range of vanadium-based compounds studied for metal–ion hybrid batteries, reflected in Table 2, further highlights the material’s remarkable versatility.

**Table 2:** Electrochemical Performance of Vanadium-Based Materials in Metal–Ion Hybrid Batteries

Vanadium-based material and counter electrode	Electrolyte	Hybrid battery ion type	Specific capacity [mAh·g^–1^]/C-rate or current density [A·g^–1^]	Potential window [V]	Ref
Na_3_V_2_(PO_4_)_2_F_3_ vs Graphite	1 M LiPF_6_ in EC/DMC (2:1 by weight)	Li–Na	115–120/0.5C	3.00–4.60	^31^
Na_3_V_2_(PO_4_)_2_F_3_ vs Li/Li^+^ (Li metal)	1 M LiPF_6_ in EC/DMC/diethyl carbonate (DEC) (1:1:1 by volume)	Li–Na	132/0.1C	1.60–4.60	^92^
Na_3_V_2_(PO_4_)_3_ vs Li/Li^+^ (Li metal)	1 M LiPF_6_ in EC/DMC/DEC (1:1:1 by volume)	Li–Na	148/0.1C	1.60–4.60	^93^
Na_3_V_2_(PO_4_)_2_F_3_@bagasse carbon vs Li/Li^+^ (Li metal)	1 M LiPF_6_ in EC/DMC	Li–Na	125/0.1C	2.30–3.70	^94^
Na_3_V_2_(PO_4_)_2_F_3_ (NVPF)/LiNi_0.8_Co_0.1_Mn_0.1_O_2_ (NCM811) composite = 8:2 (weight ratio) vs Hard Carbon (HC)	1 M LiPF_6_ in EC/DMC/EMC (1:1:1 by volume) and 0.5 M NaPF_6_ in PC/fluoroethylene carbonate (FEC) (98:2 by volume) - Vol_Na–_: Vol_Li_ ratio of 9:1.	Li–Na	147/0.1C	2.70–4.30	^95^
Na_3_V_2_(PO_4_)_2_O_2_F (NVPOF) vs mesophase carbon microbeads (MCMB)	1 M LiPF_6_ in EC/DMC/DEC	Li–Na	112.7/0.065	2.80–4.40	^96^
Na_1.5_VPO_4_F_1.5_ vs Li/Li^+^ (Li metal)	1 M LiPF_6_ in EC/DMC (2:1 by weight)	Li–Na	116/0.1C	3.00–4.60	^97^
Na_3_V_2_(PO_4_)_3_@C vs lithiated graphite	1 M LiPF_6_ and 0.5 M NaClO_4_ in EC:DEC:EMC (1:1:0.5 in volume)	Li–Na	107/0.1C	2.50–4.20	^98^
Na_3_V_2_(PO_4_)_2_F_3_@C@PTHF (polytetrahydrofuran and carbon) vs Li/Li^+^ (Li metal)	1 M LiClO_4_ and x M NaClO_4_ (x = 0, 0.002, 0.02, 0.1, 0.2, 0.4) in EC/DMC (1:1 by volume)	Li–Na	122/1C	3.00–4.50	^99^
Li_2.7_V_2.1_(PO_4_)_3_/C vs Na metal	1 M LiPF_6_ in EC/DMC	Li–Na	133/0.1	2.50–4.20	^100^
	1 M NaClO_4_ and 5 wt % FEC in EC and DMC		132/0.1		
Na_3_V_2_(PO_4_)_3_/C microflowers vs Li_4_Ti_5_O_12_	1 M LiPF_6_ in EC/DMC	Li–Na	73/0.1	1.00–3.00	^101^
Carbon-coated Na_3_V_2_(PO_4_)_3_ microflower vs Li_4_Ti_5_O_12_	1 M LiPF_6_ in EC/DEC (3:7 by volume)	Li–Na	95/0.1	1.00–2.50	^102^
Mo-Doped Na_3_V_2_(PO_4_)_3_@C nanowires vs Li_4_Ti_5_O_12_	1 M LiPF_6_ in EC/DMC/DEC (1:1:1 by volume)	Li–Na	108/0.1C	1.00–3.10	^103^
Li_3_V_2_(PO_4_)_3_ vs Zn/Zn^2+^ (Zn metal)	1 M Li_2_SO_4_ + 2 M ZnSO_4_ in H_2_O	Li–Zn	128/0.2C	0.70–2.10	^104^
Na_3_V_2_(PO_4_)_3_ vs Zn/Zn^2+^ (Zn metal)		Li–Na–Zn	96/0.2C		
Desodiated NaV_2_(PO_4_)_3_ vs Mg/Mg^2+^ (Mg metal)	0.2 M [Mg_2_Cl_2_][AlCl_4_]_2_ and 0.4 M NaAlCl_4_ in dimethoxy ethane (DME)	Na–Mg	104/1C	1.60–3.00	^105^
Partially desodiated Na_3_V_2_(PO_4_)_3_ vs Mg/Mg^2+^ (Mg metal)	0.5 M magnesium bis(trifluoromethanesulfonimide) in 1,2-DME	Na–Mg	100/0.01	0.50–2.20	^106^
Mesoporous (desodiated) Na_3_V_2_(PO_4_)_3_/C vs activated carbon (AC)	0.3 m Mg(TFSI)_2_/MeCN	Na–Mg	88.8/0.02	–0.40–0.60	^107^
Na_1.5_VPO_4.8_F_0.7_ vs Mg/Mg^2+^ (Mg metal)	2 M NaBH_4_ + 0.2 M Mg(BH_4_)_2_ in tetraglyme and 1 M NaClO_4_ in PC + β-alumina membrane	Na–Mg	110/0.2C	2.40–3.80	^30^
Na_3_V_2_(PO_4_)_3_/C vs Zn/Zn^2+^ (Zn metal)	0.5 M CH_3_COONa/Zn(CH_3_COO)_2_ in H_2_O	Na–Zn	92/0.05	0.80–1.70	^108^
Na_3_V_2_(PO_4_)_3_/C vs Zn/Zn^2+^ (Zn metal)	0.5 M Zn(CH_3_COO)_2_ in H_2_O	Na–Zn	97/0.5C	0.80–1.70	^109^
Na_3_V_2_(PO_4_)_3_/C vs Zn/Zn^2+^ (Zn metal)	0.5 M Zn(CH_3_COO)_2_ in H_2_O	Na–Zn	110/0.1C	0.60–1.60/1.80	^110^
Na_3_V_2_(PO_4_)_3_/rGO microspheres vs Zn/Zn^2+^ (Zn metal)	2 M Zn(CF_3_SO_3_)_2_ in H_2_O	Na–Zn	114/0.05	0.60- 1.80	^111^
Carbon-coated Na_3_V_2_(PO_4_)_2_F_3_ vs Carbon film functionalizing Zn	2 M Zn(CF_3_SO_3_)_2_ in H_2_O	Na–Zn	75/0.08	0.80–1.90	^112^
Na_3_V_2_(PO_4_)_3_/C/carbon nanofiber vs Zn/Zn^2+^ (Zn metal)	3 M Zn(CF_3_SO_3_)_2_ in H_2_O	Na–Zn	93/0.1	0.50–1.90	^113^
Na_3_V_2_(PO_4_)_3_ vs Zn/Zn^2+^ (Zn metal)	NaClO_4_–Zn(OTf)_2_ (8.5m:1.5m) aqueous solutions immobilized in a gel biopolymer electrolyte	Na–Zn	∼75/1C	1.10–1.60	^114^
Na_3_V_2_(PO_4_)_2_F_3_/C vs Zn/Zn^2+^ (Zn -metal)			∼70/1C	0.90–1.80	
Na_3_V_2_(PO_4_)_3_ vs Al/Al^3+^ (Al metal)	2 M NaAlCl_4_/1-ethyl-3methylimidazolium chloride (EMImC) and aluminum chloride (1–1.1)	Na–Al	96/0.2C	0.50–2.10	^115^
Na_3_VCr(PO_4_)_3_ vs Mg/Mg^2+^ (Mg metal)	0.5 M Mg(TFSI)_2_ in DME	Na–Mg	∼90/0.002	∼0.20–2.55	^116^
Hydrated V_2_O_5_ nanowires vs Zn/Zn^2+^ (Zn metal)	1 M ZnSO_4_ + 1 M Na_2_SO_4_ in H_2_O	Na–Zn	∼160/1	0.20–1.60	^117^
Zn_0.3_Na_0.43_V_2_O_5_ vs Zn/Zn^2+^ (Zn metal)			∼135/1		
Na_0.33_V_2_O_5_ vs Zn/Zn^2+^ (Zn metal)			209/1		
V_2_O_5_ nanofiber vs Mg/Mg^2+^ (Mg metal)	0.25 M all phenyl complex (APC) + 1 M anhydrous LiCl	Li–Mg	386/0.01	0.25–3.00	^37^
Porous V_2_O_5_ vs Zn/Zn^2+^ (Zn metal)	1 m Zn(CF_3_SO_3_)_2_ + 21 m LiTFSI in H_2_O	Li–Zn	242/0.05	0.20–1.60	^118^
V_2_O_5_ nanosheets vs Zn/Zn^2+^ (Zn metal)	0.5 M Zn(ClO_4_)_2_ + 1 M LiClO_4_ ^a^	Li–Zn	410/5.7	0.10–2.80	^119^
V_3_O_7_·H_2_O vs Mg/Mg^2+^ (Mg metal)	0.25 M APC + 1 M anhydrous LiCl	Li–Mg	305.4/0.025	0.50–2.00	^36^
V_3_O_7_·H_2_O vs Na-containing AC	0.5 M Mg(NO_3_)_2_ in H_2_O	Na–Mg	404/0.1	–1.10–0.20	^120^
LiV_3_O_8_@GO vs Mg/Mg^2+^ (Mg metal)	^a^APC + 1 M LiCl	Li–Mg	245.9/0.05	1.00- 2.50	^121^
Li_3_V_2_(PO_4_)_3_ vs Mg/Mg^2+^ (Mg metal)	0.4 M APC + 1 M LiCl	Li–Mg	147.8/0.05	0.05–2.00	^122^
	0.1 M Mg(BH_4_)_2_ and 1.5 M LiBH_4_ in diglyme (DG)		99.6/0.05	0.05–1.50	
NaV_3_O_8_ 1.69H_2_O vs Mg/Mg^2+^ (Mg metal)	0.4 M APC + 1 M LiCl	Li–Mg	446/0.02	0.00–2.00	^123^
β-NaV_6_O_15_ vs Mg/Mg^2+^ (Mg metal)	0.09 M Mg(BH_4_)_2_ + 0.25 M NaBH_4_ in DG	Na–Mg	125/current intensity 10 μA	0.10–1.80	^124^
Carbon coated Na_2_VTi(PO_4_)_3_ (NVTP@C) vs Mg/Mg^2+^ (Mg metal)	2.0 mmol of magnesium bis(hexamethyldisilazide) + (Mg(HMDS)_2_, was dissolved in 10 mL DG + 4.0 mmol of anhydrous AlCl_3_ + 10 mmol of NaTFSI salt	Na–Mg	168/0.05	0.10–3.00	^125^
Li–V_2_C-10 vs Mg/Mg^2+^ (Mg metal)	0.4 m APC + 0.4 m LiCl	Li–Mg	230.3/0.02	0.00–2.00	^126^
V_2_MoO_8_ vs Mg/Mg^2+^ (Mg metal)	0.4 M APC + 1 M LiCl	Li–Mg	312/0.02	0.50–2.40	^127^
VNb_9_O_25_ vs Mg/Mg^2+^ (Mg metal)	0.4 M APC + 1.5 M LiBH_4_	Li–Mg	150/0.1C	0.12–1.40	^128^
VO_2_ vs Mg/Mg^2+^ (Mg metal)	0.25 M APC + 1 M LiCl	Li–Mg	206.8/0.02	0.50–2.00	^129^
VO_2_/GO (graphene oxide) composite vs Mg/Mg^2+^ (Mg metal)	0.4 M APC + 1 M LiCl	Li–Mg	261.7/0.02	0.50–2.00	^130^
VOCl vs Mg/Mg^2+^ (Mg metal)	1.5 M (HMDS)_2_Mg-2AlCl_3_ in THF + tetraglyme (G4) (1:1 in vol.) + 0.5 M LiCl	Li–Mg	195/0.1	0.05–2.20	^131^
VS_2_ nanosheets vs Mg/Mg^2+^ (Mg metal)	0.4 M APC + 0.4 M LiCl/THF	Li–Mg	171/0.05	0.60–2.00	^132^
VS_2_ vs Mg/Mg^2+^ (Mg metal)	3 mmol Mg(HMDS)_2_ + 6 mmol AlCl_3_ in THF + 5 mmol LiTFSI	Li–Mg	236.6/0.05	0.10–2.50	^133^
	+ 5 mmol NaTFSI	Na–Mg	202.3/0.05		
	+ 5 mmol KTFSI	K–Mg	250/0.05		
VS_2_-GO vs Mg/Mg^2+^ (Mg metal)	^a^APC + 1 M LiCl	Li–Mg	235/1C	0.50–2.00	^134^
Interlayer-expanded and binder-free VS_2_ on carbon paper vs Mg/Mg^2+^ (Mg metal)	0.4 M APC + 1 M LiCl	Li–Mg	309/0.1	0.25–2.00	^135^
3D hierarchical flowerlike VS_2_ vs Mg/Mg^2+^ (Mg metal)	0.4 M APC + 1.0 M LiCl	Li–Mg	181.1/2	0.10- 2.00	^38^
VS_4_ nanodendrites vs Mg/Mg^2+^ (Mg metal)	0.4 M APC + 0.4 M LiCl	Li–Mg	∼300/0.5	0.20–2.20	^81^
			Initial cycle (668 mAh·g^–1^ (discharge) and 487 mAh·g^–1^ (charge))		
Vanadium molybdenum sulfide nanosheets vs Mg/Mg^2+^ (Mg metal)	0.4 M APC: 0.25 M BMPyrrCl	Li–Mg	211.3/0.1	0.20–2.00	^136^

a Concentration or solution is not mentioned in the publication.

The following subsections analyze the vanadium-based materials listed in Table 2, focusing on their electrochemical performance and intercalation mechanisms in various hybrid battery types.

## 4 Hybrid Batteries Based on the Intercalation of Alkali Metal Cations

### 4.1 Li–Na Hybrid Batteries

Recently, Zhang et al. reviewed the role of alkali metals (Li, Na, and K) in hybrid-ion batteries.^137^ Despite the challenges posed by the larger ionic radii of K and Na ions, their inclusion in the intercalation process can still be beneficial, as a moderate structural enlargement can widen intercalation channels and improve the ion transport in batteries.

Shen et al. showed that K doping in Na_3_V_2_(PO_4_)_3_ (NVP) widens the intercalation channels for Na ions and increases the unit cell volume, leading to a specific capacity of 114.8 mAh·g^–1^ for K_0.05_Na_2.95_V_2_(PO_4_)_3_, compared to 92.4 mAh·g^–1^ for undoped NVP at 0.5C.^138^

Conversely, Cong et al. reported that Li doping, which slightly reduces cell volume, also enhances electrochemical performance.^139^ They synthesized lithium-doped Na_2.8_Li_0.2_V_2_(PO_4_)_3_/C (NVP/C) and achieved a promising specific capacity of 116.9 mAh·g^–1^ as well as a capacity retention of 99.82% after 500 cycles, which is close to NVP’s theoretical capacity of 118 mAh·g^–1^.^104,139^ Especially, NVP and its NASICON-based variants, as shown in Table 2, are key vanadium-based material(s) for alkali metal cation intercalation in Li–Na hybrid batteries.^31,92–103^ Guo et al. employed mesophase carbon microbeads (MCMB) as the counter electrode, Na_3_V_2_(PO_4_)_2_O_2_F as the working electrode, and 1 M LiPF_6_ in EC/DMC/DEC as the electrolyte.^96^ The hybrid battery achieved 112.7 mAh·g^–1^, resulting in an energy density of 328 Wh·kg^–1^ at a current density of 0.065 A·g^–1^ (see Figure 5a).^96^ Furthermore, long-term stability measurements at 1.3 A·g^–1^ showed 86.3% capacity retention after 2000 cycles (see Figure 5b), highlighting the long-term stability of NVP in Li–Na hybrid batteries.

**Figure 5:**
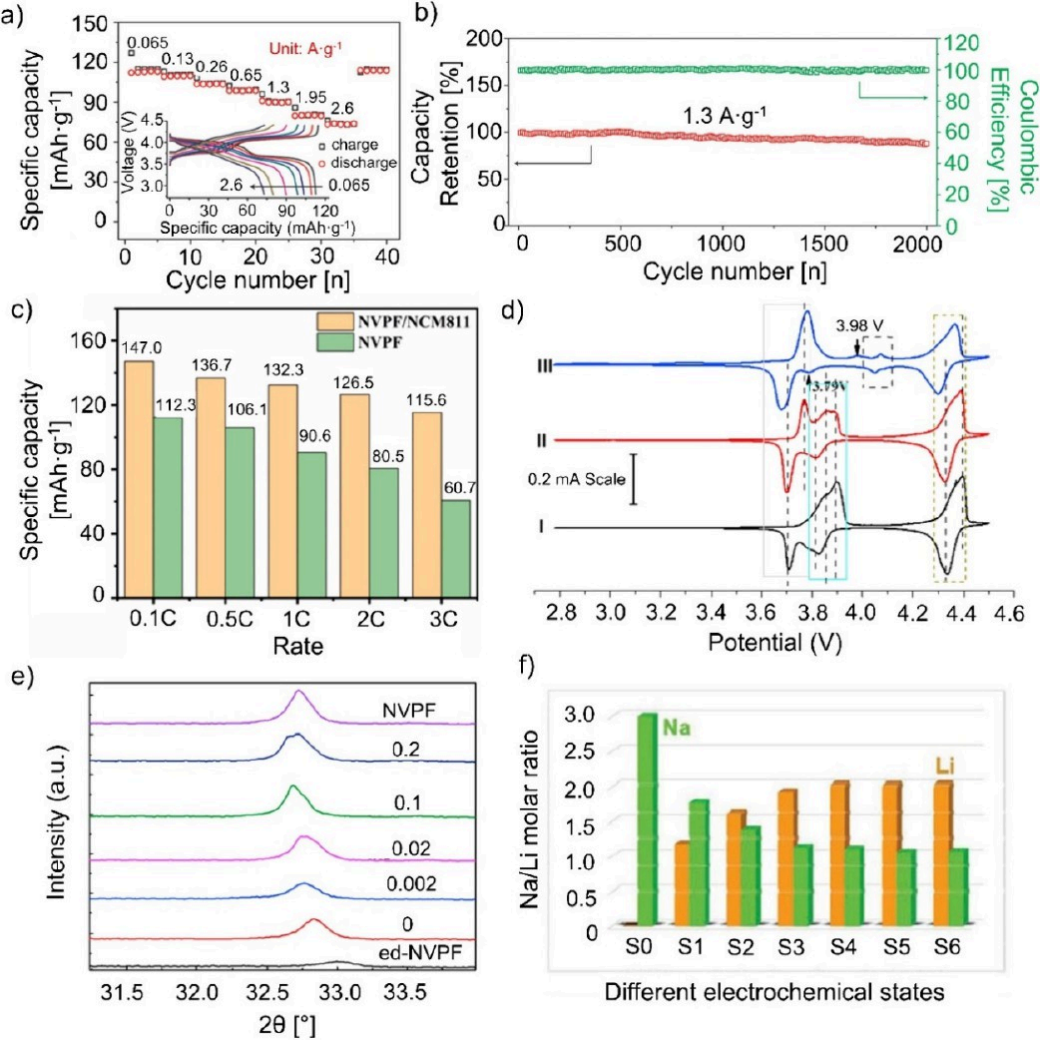
(a) Rate capability and the corresponding galvanostatic charge–discharge curves (inset) at various current densities from 0.065 to 2.6 A·g^–1^ of the assembled MCMB//NVPOF hybrid battery and (b) the cycling performances at the current density of 1.3 A·g^–1^ over 2000 cycles. Reproduced with permission from ref 96. Copyright 2018 WILEY-VCH. (c) Comparison of the rate performance between NVPF and NVPF/NCM811. Reproduced with permission from ref 95. Copyright 2025 Elsevier. (d) CV curves of the first (I), second (II), and 10th (III) cycles at 0.05 mV·s^–1^ of NVPF@C@PTHF and (e) XRD profiles of ed-NVPF@C@PTHF and NVPF@C@PTHF before and after 20 cycles in different electrolytes, magnified for (222). Reproduced with permission from ref 99. Copyright 2018 ELSEVIER. (f) Na/Li molar ratios from Mo-doped NVP in a hybrid battery at electrochemical states from S0 to S6. Reproduced with permission from ref 103. Copyright 2021 Wiley-VCH.

To enhance the electrochemical properties of Na_3_V_2_(PO_4_)_2_F_3_ (NVPF) as cathode material in a hybrid battery, Xie et al. synthesized a NVPF/LiNi_0.8_Co_0.1_Mn_0.1_O_2_ (NVPF/NCM) composite.^95^ NCM, a high-capacity and cost-effective LIB cathode,^140^ was combined with hard carbon, one of the major anode materials in SIBs^141^ and a mixed electrolyte of 1 M LiPF_6_ in EC/DMC/EMC (1:1:1 by volume) and 0.5 M NaPF_6_ in PC/fluoroethylene carbonate (FEC). In addition to incorporating FEC, which often results in an improved performance in LIBs compared to electrolytes without it,^142^ a Na salt-based electrolyte was used (to 90%) compared to previous studies. As illustrated in Figure 5c, the hybrid battery achieved a specific capacity of 147 mAh·g^–1^ at 0.1C and 136.7 mAh·g^–1^ at 0.5C, which is approximately 15% higher than the value reported by Barker et al., mainly caused by the incorporation of NCM in the composite.

In another approach, the structural and electrochemical properties of NVP and its modifications were studied using Li metal as the counter electrode. Song et al. reported specific capacities of 148 and 132 mAh·g^–1^, respectively, for NVPF at a C rate of 0.1C in the potential range of 1.60–4.60 vs Li/Li^+^.^92,93^ In addition to evaluating the electrochemical performance, they conducted a detailed investigation of the intercalation mechanism within the hybrid battery. The initial cyclovoltammetry curve differs significantly from the following cycles (second to fifth), showing a shift toward lower potentials and exhibiting a single redox couple that nearly disappears after the fifth cycle.^93^ This behavior may be attributed to simultaneous Na-ion intercalation from the cathode and Li-ion insertion from the electrolyte and the lithium metal counter electrode during the initial cycles.

Equations 1 and 2 illustrate the electrochemical reactions associated with the intercalation of Li and Na ions into NVP during the initial charge/discharge cycle.

First charge process:

1 $${\mathrm{Na}}_{3}V_{2}{\left({\mathrm{PO}}_{4}\right)}_{3}⇌{\mathrm{Na}}_{3-x}V_{2}{\left({\mathrm{PO}}_{4}\right)}_{3}+{\mathrm{xNa}}^{+}+{\mathrm{xe}}^{-}\;\left(x<2\right)$$

Following discharge process:

2 $${\mathrm{Na}}_{3-x}V_{2}{\left({\mathrm{PO}}_{4}\right)}_{3}+{\mathrm{xLi}}^{+}+{\mathrm{xe}}^{-}⇌{\mathrm{Na}}_{3-x}{\mathrm{Li}}_{x}V_{2}{\left({\mathrm{PO}}_{4}\right)}_{3}$$

During the initial charge process, Na ions are extracted from NVP, leading to the oxidation of V-sites from V^+III^ to V^+III/+IV^. During the subsequent discharge process, Li ions provided by the electrolyte and counter electrode are inserted into NVP leading to the reduction of V sites from V^+III/+IV^ to V^+III^ and a phase formation from NVP to Na_3‑x_Li_*x*_V_2_(PO_4_)_2_ (x < 2). The phase formation from NVP to NaLi_2_V_2_(PO_4_)_2_ occurs primarily during the first charge/discharge cycles.

Supporting this hypothesis, Song et al. used NVPF as cathode against Li metal and revealed a Li/V molar ratio of 0.97 after the 10th galvanostatic cycle via inductively coupled plasma (ICP) analysis.^92^ This corresponds to the composition of Li_1.94_Na_1.06_V_2_(PO_4_)_2_F_3_, indicating that Na ions were almost entirely replaced by Li ions as one Na site remains in the structure. In Figure 5d, Xiong et al. show CV measurements of the first, second, and 10th cycle of NVPF@C@PTHF illustrating how the redox peaks change during the cycling process.^99^ They further investigated the intercalation mechanism of NVPF and found that Na-ion intercalation dominates when its concentration is ≥0.2 M while the Li-ion concentration is set to 1 M. Since Li ions have a smaller ionic radius, their occupation of Na-ion sites is less stable, leading to a greater displacement from the original Na-ion site as evidenced by XRD shifts to higher angles (Figure 5e). Moreover, higher current densities favor Li-ion intercalatio compared to Na-ion intercalation due to its faster diffusion, highlighting the significant influence of the current density on hybrid battery intercalation behavior.

To demonstrate the suitability of NVP in Li–Na hybrid batteries, Liang et al. employed NVP and its modifications as cathodes in full LIB cells using, using Li_4_Ti_5_O_12_ (LTO) as the anode.^103^ This hybrid battery delivered promising specific capacities of around 108 mAh·g^–1^, while showing an inter- and deintercalation behavior similar to that using Li-metal or carbon-based materials as the counter electrode. Figure 5f illustrates the decrease of Na and the increase of Li in Mo-doped NVP across different electrochemical states (S0 = pristine Mo-doped NVP until S6 = after the 500th cycle). Analysis of the increasing Li 1s signal observed during X-ray photoelectron spectroscopy (XPS) measurements has shown that after the first cycle (S2) Li content in NVP reached 1.625, while Na content dropped to 1.383. This confirms that most of the Na has been extracted and replaced by Li already during/after the first cycle, which is consistent with previous studies lacking additional Na ions in the Li ion-containing electrolyte.

Overall, NVP/NVPF and its modifications are the most promising vanadium-based cathode materials for Li–Na hybrid batteries, exhibiting capacities close to their theoretical capacity and excellent cycling stability. The phase formation to NaLi_2_V_2_(PO_4_)_2_ occurs similarly regardless of whether Li metal, carbon-based materials or Li_4_Ti_5_O_12_ is used as the counter electrode. For reversible Na-ion intercalation and deintercalation in NVP­(F) within Li ion-containing hybrid batteries, a higher Na-ion concentration in the electrolyte is essential, as shown by Xie et al.^95^ They confirmed reversible Na-ion insertion and extraction in NVP, which were accompanied by symmetry changes during cycling. Xiong et al. concluded that in the setup of “Li-metal | 1 M LiClO_4_ | NVPF”, a concentration of ≥0.2 M NaClO_4_ is required to promote Na-ion intercalation rather than Li-ion intercalation in NVPF.^99^

Although the electrochemical properties as well as the intercalation mechanism in NVP and modifications have already been well studied, further investigation into the influence of electrolyte anions (e.g., ClO_4_^–^, PF_6_^–^, etc.) and the solvents (such as EC, DMC, DEC, FEC, PC) is of particular interest. Additionally, further vanadium-based materials such as Li_2.7_V_2.1_(PO_4_)_3_/C^100^ should be investigated as cathode material to demonstrate the potential of this material class in Li–Na hybrid batteries.

## 5 Hybrid Batteries Based on Intercalation of Alkali and Multivalent Metal Cations

### 5.1 Li–Mg Hybrid Batteries

As outlined in the Introduction, multivalent cations, such as Mg ions, Zn ions, or Al ions offer higher volumetric specific capacities due to their higher ionic charges, but their stronger polarization makes the intercalation more challenging compared to monovalent ions such as Li ions. In MIBs with Mg-metal counter electrodes, research is focused on developing electrolytes that prevent passivation and enable reversible ion exchange.^143,144^

The first reversible magnesium electrodeposition was reported in 1990 by Gregory et al., using Grignard reagents combined with aluminum halides in THF.^145^ Building on this work, Aurbach et al. successfully assembled an electrochemical cell using Mg­(AlCl_2_BuEt)_2_ in THF, with magnesium metal as the counter electrode and Mg_*x*_Mo_3_S_4_ as working electrodes.^146^ However, due to the relatively low oxidative stability (∼2.5 V) of the electrolyte, they later introduced the more stable all-phenyl complex (APC) electrolyte for MIBs in 2008.^147^ Despite growing interest in alternative magnesium-based electrolytes—such as magnesium borate-based electrolytes^148,149^ and magnesium bis­(trifluoromethane sulfonyl)­imide Mg­(TFSI)_2_ ^150^ in acetonitrile or diglyme (DG)—the APC electrolyte remains widely used for vanadium-based cathodes and Mg-metal anodes. The APC electrolyte involves the dissolution of AlCl_3_ and C_6_H_5_MgCl (PhMgCl) in THF at a specific molar ratio.^147^ A Lewis acid–base reaction occurs between the two components, with AlCl_3_ acting as the Lewis acid and PhMgCl as the Lewis base. Although the APC electrolyte generally enables the use of Mg metal as a counter electrode, the electrochemical performance remains limited, particularly with vanadium-based materials due to the limited reachable potential. Besides, there is the possibility of side reactions with stainless steel current collectors, which can be reduced by using molybdenum or graphitic-based current collectors;^151,152^ the voltage in such battery cells is general lower than 3 V. In this context, the use of Li salts in magnesium electrolytes has enabled the successful application of vanadium-based materials in MIBs with Mg metal as counter electrode, while keeping the voltage <3.0 V as shown in Table 2.^36–38,81,121–123,126–136^ Rashad et al. established promising electrochemical properties for Li_3_V_2_(PO_4_)_3_ as a cathode material in a Li–Mg hybrid battery, using a 0.4 M APC + 1.0 M LiCl in THF (APC-LiCl) and 0.1 M Mg­(BH_4_)_2_ and 1.5 M LiBH_4_ in DG (Mg/Li-BH) as electrolytes.^122^ The rate capability measurements indicate higher specific capacities with the APC-LiCl electrolyte, which achieves approximately 30–50% higher specific capacities for all methods (e.g., 147.8 mAh·g^–1^) compared to the Mg/Li-BH electrolyte (e.g., 99.6 mAh·g^–1^) at 50 mA·g^–1^. Similar electrochemical results were obtained by other vanadium-based compounds like V_2_O_5_ with 167 mAh·g^–1^ at 100 mA·g^–^,^137^ V_3_O_7_·H_2_O with 305.4 mAh·g^–1^ at 25 mA·g^–1^ (Figure 6a),^36^ LiV_3_O_8_@GO with 245.9 mAh·g^–1^ at 50 mA g^–1^,^121^ NaV_3_O_8_·1.69H_2_O with 446 mAh·g^–1^ at 20 mA·g^–1^ (Figure 6b),^123^ Li–V_2_C with 230.3 mAh·g^–1^ at 20 mA·g^–1^ (Figure 6c),^126^ V_2_MoO_8_ with 312 mAh·g^–1^ at 20 mA·g^–1^ (Figure 6d),^127^ VNb_9_O_25_ with 150 mAh·g^–1^ at 0.1C,^128^ VO_2_/GO with 261.7 mAh·g^–1^ at 20 mA·g^–1^ (Figure 6e),^130^ VS_2_ with 309 mAh·g^–1^ at 100 mA·g^–1^ (Figure 6f),^135^ and VS_4_ with 300 mAh·g^–1^ at 500 mA·g^–1^ (Figure 6g).^81^

**Figure 6:**
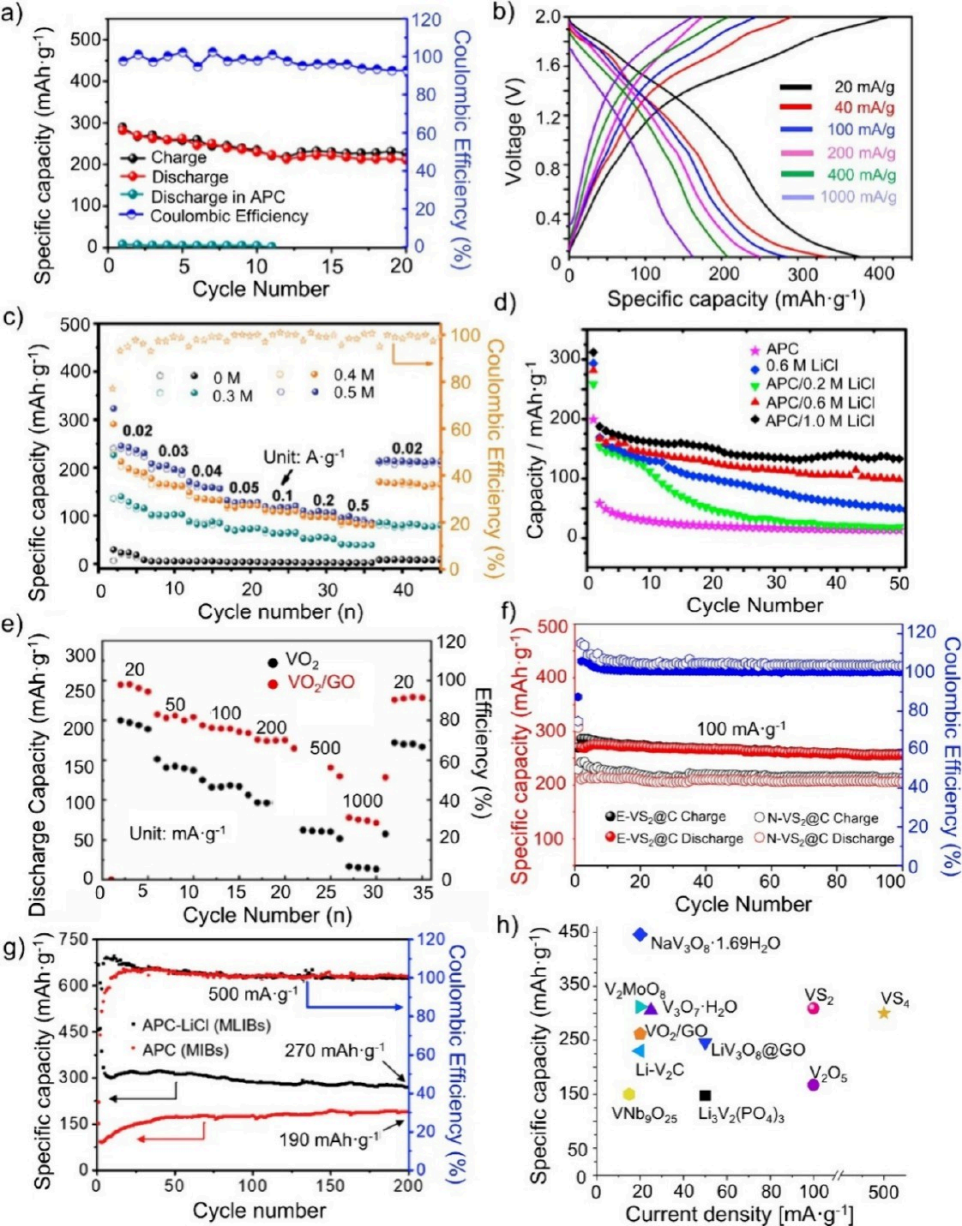
Electrochemical performance of several vanadium-based materials in Li–Mg hybrid batteries. Used as cathode material vs Mg metal in an APC Mg–Li containing electrolyte: (a) V_3_O_7_·H_2_O. Reproduced with permission from ref 36. Copyright 2017 ACS. (b) NaV_3_O_8_·1.69H_2_O. Reproduced with permission from ref 123. Copyright 2018 ACS. (c) Li–V_2_C. Reproduced with permission from ref 126. Copyright 2020 Wiley-VCH. (d) V_2_MoO_8_. Reproduced with permission from ref 127. Copyright 2017 Elsevier. (e) VO_2_/GO. Reproduced with permission from ref 130. Copyright 2022 Elsevier. (f) Expanded VS_2_. Reproduced with permission from ref 135. Copyright 2020 Elsevier. (g) VS_4_. Reproduced with permission from ref 81. Copyright 2019 Elsevier. (h) Overview of the specific capacity of vanadium-based cathode materials depending on the current density.

Figure 6h summarizes the listed vanadium-based materials in Li–Mg hybrid batteries using an APC-LiCl electrolyte and Mg metal as the counter electrode. At low current densities <30 mA·g^–1^, NaV_3_O_8_·1.69H_2_O exhibits the highest specific capacity. At higher current densities >100 mA·g^–1^, vanadium sulfides, (VS_2_ and VS_4_), show promising specific capacities of 300 mAh·g^–1^ at 100 and 500 mA·g^–1^, respectively. The improved electrochemical performance compared to other vanadium-based materials can be attributed to larger interlayer spaces (e.g., 5.76 Å for VS_2_ ^153^ compared to 4.37 Å for V_2_O_5_ ^154^) and higher electrical conductivities (e.g., 5·10^–2^ S·m^–1^ for VS_2_ ^90^ compared to 1·10^–4^ S·m^–1^ for NVP^155^). Rashed et al. described the overall intercalation process in the “Mg-metal | APC-LiCl | NVP” battery cell during the discharge process corresponding to eqs 3 and 4.^122^

Anode:

3 $$2\mathrm{Mg}+3{\mathrm{Cl}}^{-}+6\left(C_{4}H_{8}O\right){\left[{\left(\mu -\mathrm{Cl}\right)}_{3}{\mathrm{Mg}}_{2}{\left(C_{4}H_{8}O\right)}_{6}\right]}^{+}+4e^{-}$$

Cathode:

4 $${\mathrm{LiV}}_{2}{\left({\mathrm{PO}}_{4}\right)}_{3}+2{\mathrm{Li}}^{+}+2e^{-}⇌{\mathrm{Li}}_{3}V_{2}{\left({\mathrm{PO}}_{4}\right)}_{3}$$

In this case, Mg-ion inter- and deintercalation occurs at the anode, while Li ions undergo inter- and deintercalation at the cathode, reminiscent of the behavior of a Daniel type hybrid battery (see Figure 2a).

A similar reaction mechanism for the cathode was expressed by Diem et al. for V_2_O_5_ (eq 5),^37^ Tang et al. for V_3_O_7_·H_2_O (eq 6),^36^ and Rashad et al. for NaV_3_O_8_·1.69H_2_O (eq 7).^123^

5 $$V_{2}O_{5}+{\mathrm{Li}}^{+}+e^{-}⇌{\mathrm{LiV}}_{2}O_{5}$$

6 $$V_{3}O_{7}\cdot H_{2}O+{\mathrm{Li}}^{+}+e^{-}⇌{\mathrm{LiV}}_{3}O_{7}\cdot H_{2}O$$

7 $${\mathrm{NaV}}_{3}O_{8}\cdot 1.69H_{2}O+{\mathrm{Li}}^{+}+e^{-}⇌{\mathrm{NaLixV}}_{3}O_{8}\cdot 1.69H_{2}O$$

Interestingly, Liu et al. and Wang et al. described a different behavior for Li–V_2_C (eqs 8 and 9)^126^ and VS_4_ (eqs 10 and 11),^135^ respectively. They describe a cointercalation of Li and Mg ions in the cathode material, denoted as cointercalation type as shown in Figure 2b.

8 $$V_{2}C+0.15{\mathrm{Li}}^{+}+0.15e^{-}\to {\mathrm{Li}}_{0.15}V_{2}C\;\left(\mathrm{prelithiation step}\right)$$

9 $${\mathrm{Li}}_{0.15}V_{2}C+0.44{\mathrm{Mg}}^{2+}+0.35{\mathrm{Li}}^{+}+1.23e^{-}⇌{\mathrm{Mg}}_{0.44}{\mathrm{Li}}_{0.5}V_{2}C$$

10 $${\mathrm{VS}}_{4}+0.77{\mathrm{Mg}}^{2+}+0.2{\mathrm{Li}}^{+}+1.75e^{-}\to {\mathrm{Mg}}_{0.77}{\mathrm{Li}}_{0.21}{\mathrm{VS}}_{4}$$

11 $${\mathrm{Mg}}_{0.77}{\mathrm{Li}}_{0.21}{\mathrm{VS}}_{4}+0.63{\mathrm{Mg}}^{2+}+0.49{\mathrm{Li}}^{+}+1.75e^{-}⇌{\mathrm{Mg}}_{1.4}{\mathrm{Li}}_{0.7}{\mathrm{VS}}_{4}$$

The reaction on the anode for those compounds is similar to that in eq 3, or simplified as that in eq 12, highlighting that there is only Mg-ion inter- and deintercalation on the anode.

Anode:

12 $$\mathrm{Mg}⇌{\mathrm{Mg}}^{2+}+2e^{-}$$

This raises the question of whether different vanadium-based cathodes in the “Mg-metal | APC-LiCl | vanadium-based cathode” setup follow distinct intercalation mechanisms, either Daniel-type or cointercalation type. Variations in the specific capacity may explain this different intercalation behavior, as Mg ions exhibit slower insertion kinetics than Li ions due to their divalent charge, potentially limiting Mg-ion intercalation at low capacities or current densities that are too high current densities. However, NaV_3_O_8_·1.69H_2_O exhibits a high specific capacity of 446 mAh·g^–1^ at current densities below 30 mA·g^–1^ with no detectable Mg in the cathode.^123^ In contrast, Li–V_2_C achieves a specific capacity of 230.3 mAh·g^–1^ at 20 mA·g^–1^ and forms Mg_0.44_Li_0.5_V_2_C after the discharge process, indicating nearly equal Mg- and Li-content in the cathode.^126^

Analyzing the various results in more detail, it becomes evident that varying Li-salt concentrations in the electrolyte has a beneficial impact on the electrochemical properties (see Figure 6c, d).^126,127^ This finding is also seen for other materials in Li–Mg hybrid batteries, such as Mo_6_S_8_ vs Mg/Mg^2+^, indicating that higher Li-ion activity favors Li intercalation.^156^ Notably, V_2_MoO_8_ (0.4 M APC + 1.0 M LiCl)^127^ and Li–V_2_C (0.4 M APC + 0.4 M LiCl)^126^ use similar or lower quantities of Li in the electrolyte and still incorporate Mg in the electrode compared to NaV_3_O_8_·1.69H_2_O (0.4 M APC + 1.0 M LiCl).^123^ This suggests that the Li-ion concentration in the electrolyte is not the only criteria of the intercalation mechanism.^45^ The energy barrier for Mg-ion diffusion within the cathode material also plays a critical role, and this barrier varies with factors such as crystal structure, phase, and modifications like substitution or proton/water insertion.^157–160^ Figure 7a and b shows the activation barriers for Mg diffusion in α-V_2_O_5_ and δ-V_2_O_5_ calculated by the nudged elastic band (NEB) method.^159^ Sai Gautam et al. demonstrate that the migration energy needed for Mg-ion diffusion is much lower for δ-V_2_O_5_ (∼600–760 meV) compared to the α-V_2_O_5_ (∼975–1120 meV). Rong et al. further verified that the migration barrier for Li-ions diffusion in V_2_O_5_ is significantly smaller (∼150–200 meV) compared to multivalent ions as shown in Figure 7c.^158^ Additionally, the insertion of H^+^ lowers the migration energy and facilitate Mg-ion diffusion as reported by Ni et al.^157^ They conclude that H^+^ partly compensates and shields the interaction of the Mg ions with the negatively charged vanadyl oxygen of the α-V_2_O_5_ layer. Furthermore, H_2_V_2_O_5_ provides an expansion of the crystal structure compared to unmodified V_2_O_5_, which also enlarges the diffusion tunnels for Mg ions. They could decrease the activation energy needed for Mg-ion diffusion in α-V_2_O_5_ from 1280 to 560 meV. In a similar observation, although Diem et al. synthesized V_2_O_5_ with an expanded interlayer distance of 9.88 Å^37^ compared to the interlayer space of 4.37 Å^154^ of pure V_2_O_5_, they only reported reversible inter- and deintercalation of Li ions into V_2_O_5_.^37^ This indicates that, despite the expanded structure, Mg-ion diffusion in V_2_O_5_ combined with the large amount of Li ions in the electrolyte remained limited.

**Figure 7:**
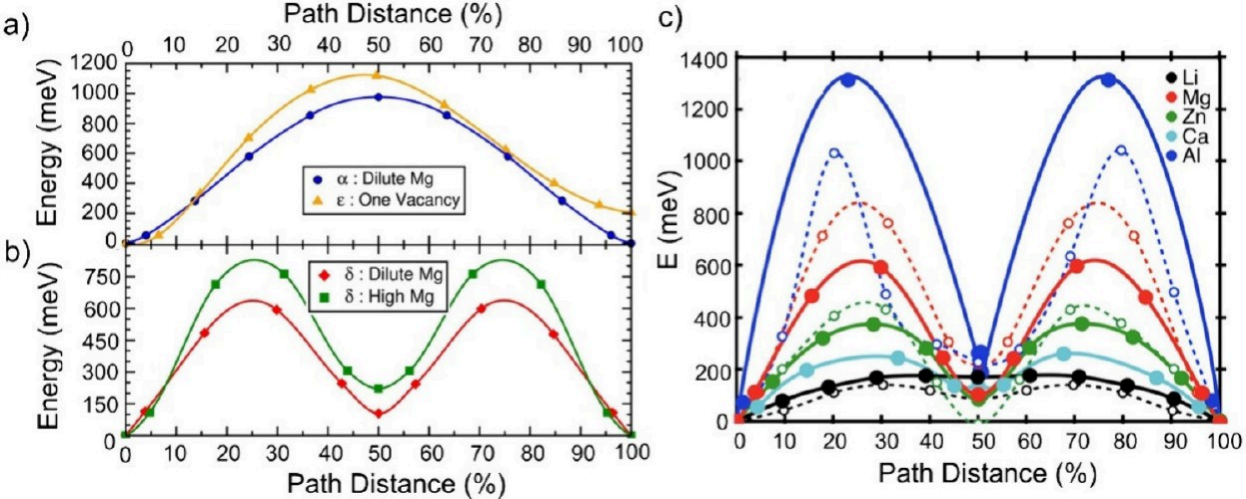
Activation barriers (a) for Mg diffusion in select limiting cases in α-V_2_O_5_ and (b) for Mg diffusion in δ-V_2_O_5_ calculated through the NEB method. Reproduced with permission from ref 159. Copyright 2015 ACS. (c) Li^+^, Mg^2+^, Zn^2+^, Ca^2+^, and Al^3+^ in the δ−V_2_O_5_ structure. Reproduced with permission from ref 158. Copyright 2015 ACS.

Compared to V_2_O_5_, other vanadium oxides like VO_2_ (i.e., VO_2_(B)^161^ or VO_2_(R)^160^) show lower Mg-ion migration barriers, facilitating the intercalation of Li and Mg ions in Li–Mg hybrid batteries.^130,160^ Kou et al. confirmed reversible Mg-ion inter- and deintercalation in VO_2_/GO via XPS measurements.^130^ Conversely, Pei et al. observed no cointercalation of Li and Mg ions in VO_2_, possibly due to a lower concentration of the APC electrolyte (0.25 M vs 0.4 M) or the absence of GO compared to the study of Kou et al.^129,130^

Compared to oxides, sulfides are more polarizable, and S^2–^ has a larger ionic radius as well as a lower electronegativity compared to O^2–^, which leads to smaller migration barriers (e.g., for Mg-ions).^27,162^ Consequently, Li-/Mg-ion cointercalation might be more favorable in sulfides. For instance, Wang et al. concluded that the specific capacity reached in VS_4_ as cathode material in a Li–Mg hybrid battery is primarily attributed to Mg ions, while Li ions contribute more to the reaction kinetics.^81^ However, this intercalation mechanism is not visible for all vanadium sulfides as Zhang et al. and Hu et al. reported that the cointercalation process of Li and Mg ions into VS_2_ is not (fully) reversible and leads to a collapse of VS_2_.^38,133^

In summary, Li–Mg hybrid batteries with vanadium-based cathodes show promising specific capacities of 150–446 mAh·g^–1^, comparable to those of LIBs. The intercalation mechanism in these materials varies, influenced by the Li-ion concentration in the electrolyte, the energy barrier needed for Li/Mg-ion diffusion, and the structure, phase, and interlayer space of the cathode material. Depending on these parameters, the intercalation mechanisms follow either only a Li-ion (Daniel type, Figure 2a) or Li-/Mg-ion (cointercalation, Figure 2b) insertion into the cathode. On the anode, only Mg ions undergo reversible inter- and deintercalation on Mg metal even at high concentrations of Li in the electrolyte.

### 5.2 Na–Mg Hybrid Batteries

In addition to Li–Mg hybrid batteries, Na–Mg hybrid batteries have gained interest due to the ability of Na ions to induce larger interlayer expansion than Li ions which often results in higher specific capacities and energy densities, while Mg-ion intercalation enhances the specific capacity compared to SIBs. Figure 8 shows the performance of vanadium-based materials in the Na–Mg hybrid batteries. The most studied vanadium-based cathode in Na–Mg hybrid batteries is NVP leading to specific capacities of 88.8 mAh·g^–1^ at 20 mA·g^–1^ (Figure 8a),^107^ 100 mAh·g^–1^ at 10 mA·g^–1^ (Figure 8b),^106^ and 104 mAh·g^–1^ at 1C (Figure 8c),^105^ which are comparable to NVP in SIBs.^138^ Despite all studies observing partial desodiation, they report different intercalation mechanisms. Li et al. observed exclusive Na-ion intercalation in NVP, likely due to the strong Coulombic interactions between Mg ions and desodiated NVP (NaV_2_(PO_4_)_3_).^105^

**Figure 8:**
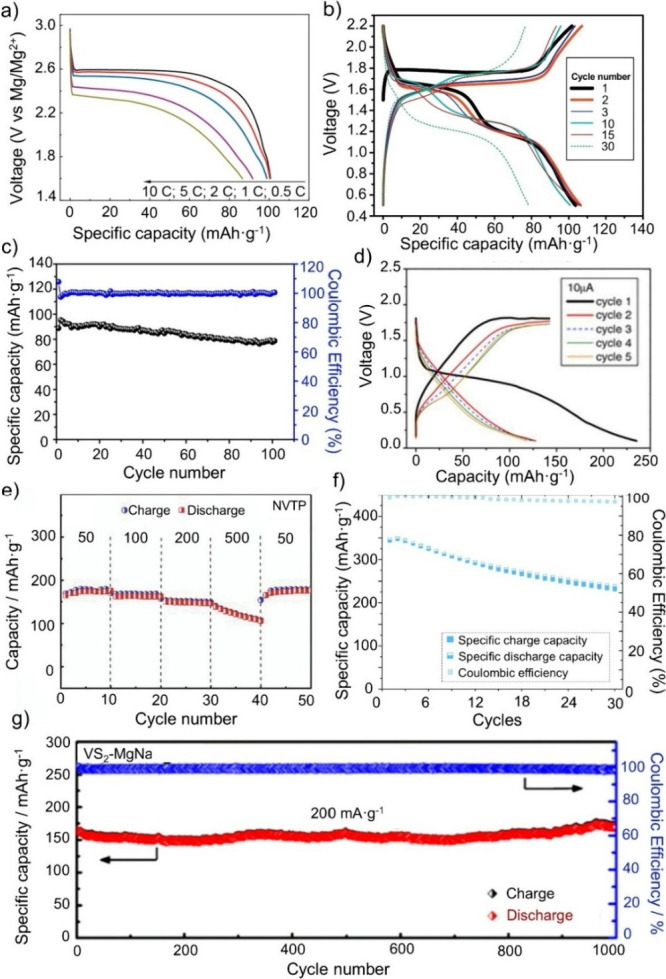
Electrochemical performance of vanadium-based materials in Na–Mg hybrid batteries. Used as cathode material: (a) NaV_2_(PO_4_)_3_. Reproduced with permission from ref 105. Copyright 2017 Elsevier. (b) NaV_2_(PO_4_)_3_. Reproduced with permission from ref 106. Copyright 2017 Elsevier. (c) NaV_2_(PO_4_)_3_/C. Reproduced with permission from ref 107. Copyright 2017 Wiley-VCH. (d) β-NaV_6_O_15_. Reproduced with permission from ref 124. Copyright 2016 The Electrochemical Society. (e) Na_2_VTi­(PO_4_)_3_. Reproduced with permission from ref 125. Copyright 2020 Elsevier. f) V_3_O_7_·H_2_O. Reproduced with permission from ref 120. Copyright 2025 ACS. (g) VS_2_;.Reproduced with permission from ref 133. Copyright 2021 ACS.

In contrast, Cabello et al. and Zeng et al. describe a cointercalation of Na and Mg ions in NVP, which was verified by theoretical calculations and experimental methods (inductively coupled plasma–atomic emission spectrometry (ICP-AES) and electron probe microanalysis (EPMA)).^106,107^

These differences are likely due to variations in the electrolytes, concentration, counter electrodes, and voltage windows. Li et al. used 0.2 M [Mg_2_Cl_2_]­[AlCl_4_]_2_ and 0.4 M NaAlCl_4_ in dimethoxy ethane (DME)^105^ favoring Na-ion intercalation in NVP. Meanwhile, Cabello et al. and Zeng et al. used 0.5 M Mg­(TFSI)_2_ in 1,2-DME^106^ and 0.3 m Mg­(TFSI)_2_/MeCN^107^ providing a Na-/Mg-ion cointercalation in NVP.

The exclusive Na-ion intercalation reported by Li et al. may stem from the higher Na-ion concentration in the electrolyte, whereas in other studies Na ions originated solely from desodiated NVP, resulting in lower overall Na-ion availability.^105–107^

Furthermore, Cabello et al. reported that first Na ions and second Mg ions are inserted into NVP, indicating that a higher Na-ion content in the electrolyte/battery favors solely intercalation of Na-ions (Daniel type) over a cointercalation.^106^ They further indicate that it is more difficult to release and intercalate Mg ions in NVP from the electrolyte solution (which contains MgCl^+^) compared to other electrolytes such as Mg­(BH_4_)_2_ in DG, highlighting that the solution in the electrolyte has a critical role in the electrochemical behavior.

Rubio et al. established a battery using Na_3_VCr­(PO_4_)_3_ as cathode material vs Mg metal in the chloride-free electrolyte Mg­(TFSI)_2_ in DME.^116^ This battery resulted in a specific capacity of ∼90 mAh·g^–1^ at 2 mA·g^–1^ at −15 °C. Furthermore, ICP-AES analysis confirmed reversible Na-/Mg-ion intercalation, with a fully discharged Mg cell (after Na-ion removal) leading to a Na:Mg:V:Cr:P ratio of 1.53:0.68:0.99:0.98:3.

Figure 8d–g shows electrochemical results of β-NaV_6_O_15_,^124^ Na_2_VTi­(PO_4_)_3_,^125^ V_3_O_7_·H_2_O,^120^ and VS_2_ ^133^ as electrode material in Na–Mg hybrid batteries, respectively.

For β-NaV_6_O_15_, Cabello et al. achieved a specific capacity of 125 mAh·g^–1^ at 10 μA using 0.09 M Mg­(BH_4_)_2_ and 0.25 M NaBH_4_ in DG as the electrolyte (see Figure 8d).^124^ They do not only indicate that Na and Mg ions intercalated in NaV_6_O_15_, but also a (co)­deposition of Na ions on the counter electrode occurs, which was not observed for Li–Mg hybrid batteries as discussed before.

Zhang et al. analyzed carbon-coated Na_2_VTi­(PO_4_)_3_ (NVTP@C) as the cathode, 2.0 mmol of Mg­(HMDS)_2_, in 10 mL DG, 4.0 mmol of anhydrous AlCl_3_, and 10 mmol of NaTFSI salt as the electrolyte, and Mg metal as the counter electrode.^125^ With this configuration, they reached specific capacities of 168, 162, 145, and 125 mAh·g^–1^ at current densities of 50, 100, 200, and 500 mA·g^–1^, respectively, as displayed in Figure 8e. Similar to Li et al. using NVP as cathode material in a Na–Mg hybrid battery, the intercalation mechanism depends solely on Na ions rather than Mg ions.^105,125^ This is probably due to the sufficient Na content in the electrolyte, which might intercalate first fully into NVTP@C before Mg ions can cointercalate into the structure.

In a different setup, Söllinger et al. showed that V_3_O_7_·H_2_O can reversibly intercalate Na and Mg ions in the structure using 0.5 M Mg­(NO_3_)_2_ in H_2_O as the electrolyte and a Na-containing AC pellet.^120^ They reached an initial specific capacity of 404 mAh·g^–1^ at 0.1 A·g^–1^ in an aqueous electrolyte (see Figure 8f). Unlike the findings of Cabello et al. for NVP, operando XRD measurements indicated that first mainly Mg ions intercalate into the structure, followed by Na ions. This deviation may arise from the use of an aqueous rather than an organic electrolyte, as previous studies have demonstrated that the sluggish Mg-ion intercalation can be mitigated in the presence of water,^163,164^ as well as variations in electrode materials, Na ion concentration, and electrochemical parameters.

Hu et al. investigated VS_2_ as cathode material in Na–Mg hybrid batteries reaching a specific capacity of 202.3 mAh·g^–1^ (Figure 8g).^133^ Using an electrolyte composed of 3 mmol of Mg­(HMDS)_2_, 6 mmol of AlCl_3_ in THF, and 5 mmol of NaTFSI, they confirmed reversible Na-/Mg-ion cointercalation. Compared to the group’s earlier findings on Li–Mg hybrid batteries, the more stable Na-/Mg-ion cointercalation in VS_2_ is likely due to a balanced Na-/Mg-ion ratio in the electrolyte and the favorable Mg-ion diffusion properties in vanadium sulfides. Hu et al. concluded that this might be caused by the larger ionic size of Na ions (1.02 Å)^4^ compared to Li ions (0.76 Å),^4^ which additionally expands the crystal structure and enlarges the intercalation channels.^133^ This is illustrated in more detail in Figure 9a for Li-/Mg-ion intercalation and Figure 9b Na-/Mg-ion intercalation in VS_2_.

**Figure 9:**
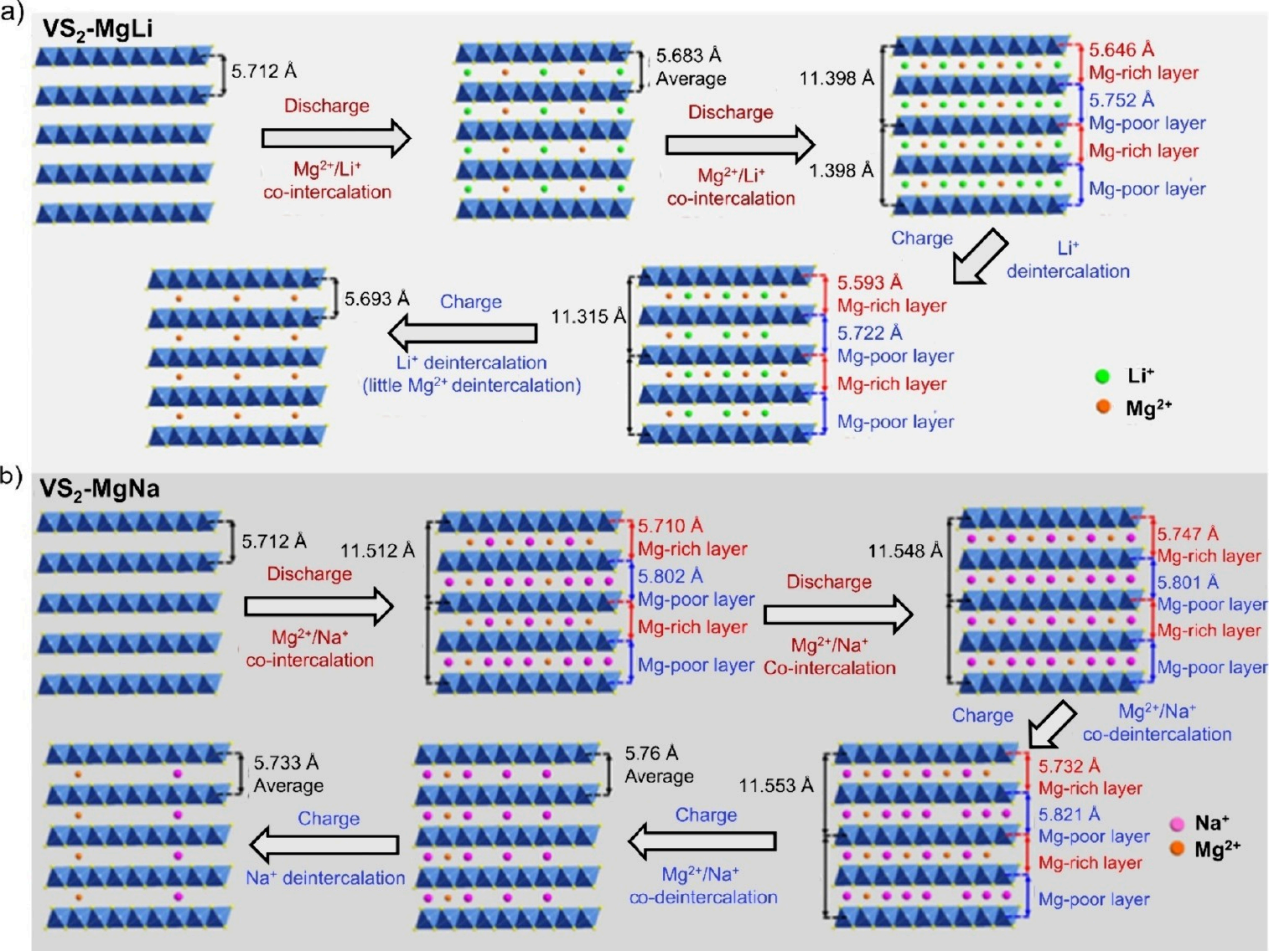
Crystal structure evolutions of VS_2_ in the (a) Mg^2+^/Li^+^ hybrid cell and (b) Mg^2+^/Na^+^ hybrid cell. Reproduced with permission from ref 133. Copyright 2021 ACS.

In summary, Na–Mg hybrid battery studies with vanadium-based cathodes have so far focused on materials such as NaV_2_(PO_4_)_3_, Na_3_VCr­(PO_4_)_3_, V_3_O_7_·H_2_O, β-NaV_6_O_15_, Na_2_VTi­(PO_4_)_3_, and VS_2_. They exhibit specific capacities ranging from 88.8 to 404 mAh·g^–1^ (using Mg metal or AC as a counter electrode). These specific capacities are generally lower compared to the values in Li–Mg hybrid batteries, likely due to the larger ionic radius of Na ions compared to Li ions, which limits the specific capacity as also seen in LIBs and SIBs for numerous electrode materials. Na–Mg hybrid batteries may favor a cointercalation of Mg and Na ions more than Mg and Li ions in the same electrode materials. Na ions result in larger interlayer expansion and have a migration barrier closer to Mg ions, promoting simultaneous insertion and improving compatibility with Mg-ion intercalation.

An additional observation in Na–Mg hybrid batteries is the possible reaction between Na ions and the Mg metal anode, as reported by Cabello et al.^124^ The finding highlights the need for further investigation of the anode in hybrid batteries to prevent irreversible side reactions that may affect the electrochemical performance.

### 5.3 Na–Zn Hybrid Batteries

Although Zn and Mg ions share similar properties, such as a divalent charge and comparable ionic radii (0.74 Å for Zn^2+^ and 0.72 Å for Mg^2+^), they also show differences, e.g., in their potentials vs SHE, namely, −0.76 V for Zn/Zn^2+^ and −2.37 V for Mg/Mg^2+^ (see Table 1). The higher potential of Zn/Zn^2+^ is beneficial for aqueous electrolytes, where the operating voltage window is limited. In addition, Zn ions exhibit a better reversible inter- and deintercalation on Zn metal compared with Mg ions on Mg metal, enabling the use of a broader range of (simple) electrolytes. NVP is often used as a cathode material in Na–Zn hybrid batteries, showing specific capacities of 92 mAh·g^–1^ at 50 mA·g^–1^ (see Figure 10a),^108^ 97 mAh·g^–1^ at 0.5C (see Figure 10b),^109^ ∼110 mAh·g^–1^ at 0.1C (see Figure 10c),^110^ and 114 mAh·g^–1^ at 50 mA·g^–1^ (see Figure 10d).^111^ In addition, carbon-coated NVPF was measured against carbon film functionalizing Zn, leading to a specific capacity of 75 mAh·g^–1^ at 80 mA·g^–1^ (see Figure 10e).^112^

**Figure 10:**
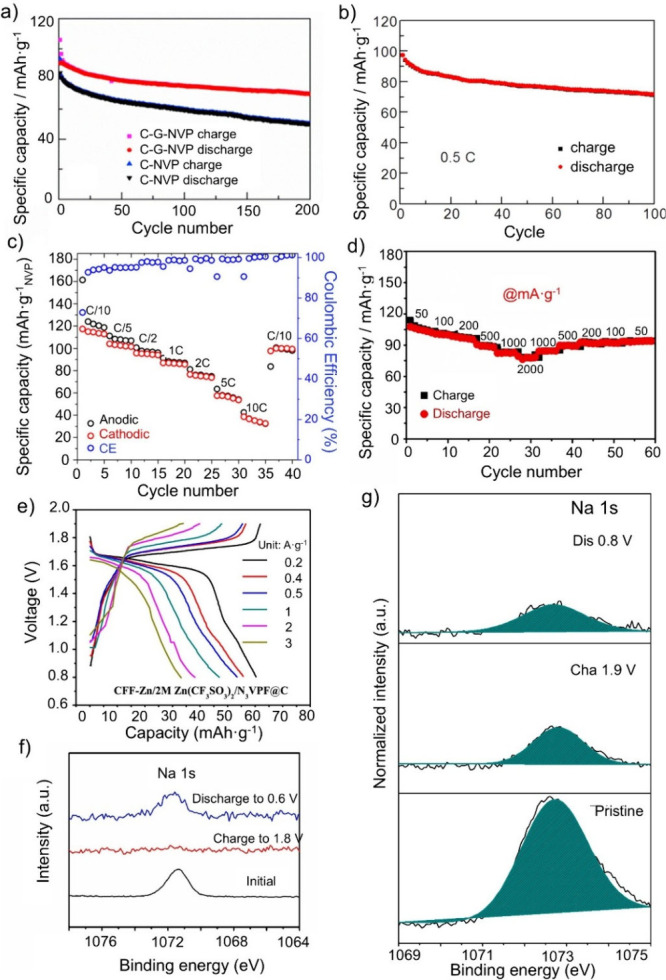
Electrochemical performance of vanadium-based materials in Na–Zn hybrid batteries: (a) NVP/C vs Zn/Zn^2+^;. Reproduced with permission from ref 108. Copyright 2016 Elsevier. (b) NVP vs Zn/Zn^2+^. Reproduced with permission from ref 109. Copyright 2016 Elsevier. (c) NVP vs Zn/Zn^2+^. Reproduced with permission from ref 110. Copyright 2020 ACS. (d) NVP/rGO microspheres vs Zn/Zn^2+^. Reproduced with permission from ref 111. Copyright 2019 Elsevier. (e) Carbon-coated NVPF vs carbon film functionalizing Zn. Reproduced with permission from ref 112. Copyright 2018 Elsevier. XPS spectra of Na 1s in its original, charged, and discharged states of (f) NVP@rGO. Reproduced with permission from ref 111. Copyright 2019 Elsevier. (g) Carbon-coated NVPF. Reproduced with permission from ref 112. Copyright 2018 Elsevier.

Li et al. studied NVP in 0.5 M Zn­(CH_3_COO)_2_ with^108^ and without^109^ 0.5 M CH_3_COONa. Without the additional Na ions in the electrolyte, inter- and deintercalation after full desodiation is dominated solely by Zn ions, whereas with Na ions reversible Na-ion intercalation occurs in NVP. They further showed that the pH value in the electrolyte strongly affects the performance since NVP dissolves more readily in an acidic electrolyte, causing significant capacity fading in sulfate and nitrate electrolytes, while partially neutral electrolytes mitigate this effect.^108^

Ko et al. used a similar setup as Li et al. with NVP as cathode vs Zn/Zn^2+^ and 0.5 M Zn­(CH_3_COO)_2_ as electrolyte, leading to ∼110 mAh·g^–1^ at 0.1C.^110^ They investigated the structural changes of NVP during the first charge–discharge cycle in more detail and verified that a quasi-two-stage Na- and Zn-ion insertion mechanism is responsible for the electrochemical behavior of NVP. After the complete desodiation of NVP, a reversible intercalation of solely Zn ions into the NVP structure occurred, similar to that in previous reports.

Hu et al. showed that the cointercalation of Na and Zn ions in NVP can be improved in Na–Zn hybrid batteries using 2.0 M Zn­(CF_3_SO_3_)_2_ as the electrolyte.^111^ In addition to a promising specific capacity of 114 mAh·g^–1^ at 50 mA·g^–1^, XPS measurements after the discharge and charge process reveal the reversible intercalation of Na ions in NVP (see Figure 10f), which was not observed in previous studies without the inclusion of a Na-containing electrolyte.^108–110^ In this case, the differences in the intercalation mechanism might mainly be caused by the different electrolytes, as Zn­(CF_3_SO_3_)_2_ has a higher ionic conductivity and lower polarization compared to Zn­(CH_3_COO)_2_, which might be beneficial.^165^ However, using Zn­(CF_3_SO_3_)_2_ as the electrolyte in a Na–Zn hybrid battery does not always lead to a cointercalation of Na/Zn ions. In another approach, Li et al. used 2.0 M Zn­(CF_3_SO_3_)_2_ as the electrolyte and NVPF as the cathode material measured against a carbon film-functionalizing Zn.^112^ Although not all experimental details coincide, they used the same electrolyte as Hu et al. and did not observe the high reversibility of Na ions into NVPF as shown in Figure 10g.^111,112^ Furthermore, Xu et al. also did not report a reversible cointercalation of Na and Zn ions using NVP/C/carbon nanofiber vs Zn/Zn^2+^ (Zn metal) in a 3 M Zn­(CF_3_SO_3_)_2_ electrolyte.^113^ The different observations can be caused by the differences in the electrode materials, different concentrations in the electrolyte, the used potential window or current density, and additional factors.

Further vanadium-based materials explored for Na–Zn hybrid batteries are V_2_O_5_ as well as Zn_0.3_Na_0.43_V_2_O_5_ and Na_0.33_V_2_O_5_. Han et al. investigated the electrochemical performance of these compounds against Zn metal in aqueous electrolytes containing 2 M ZnSO_4_, 1 M Na_2_SO_4_, or a combination of 1 M ZnSO_4_+1 M Na_2_SO_4_.^117^ Figure 11a–c shows the CV measurements of hydrated V_2_O_5_ in the mentioned electrolytes, where more distinct redox peaks are visible if the mixed electrolyte is used, compared to the individual electrolytes. An enhanced redox activity resulting in two redox peaks is observed for the 1 M ZnSO_4_+1 M Na_2_SO_4_ electrolyte, which is attributed to the inter- and deintercalation of both Na and Zn ions into the V_2_O_5_ structure. This cointercalation was further confirmed by XRD and inductively coupled plasma optical emission spectroscopy (ICP-OES) measurements.

**Figure 11:**
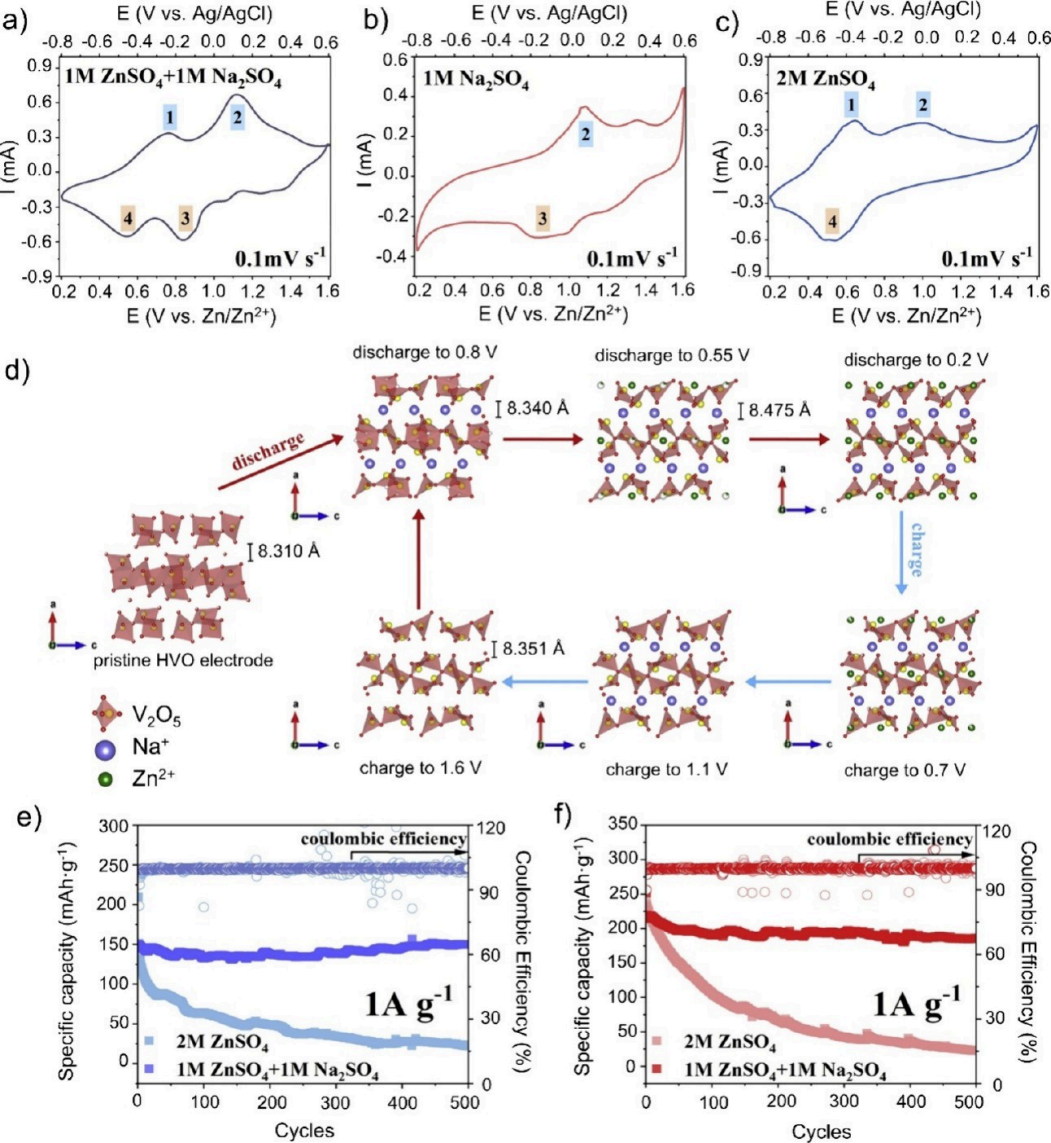
Cyclovoltammetry measurement of hydrated V_2_O_5_ (HVO) at a scan rate of 0.1 mV·s^–1^ in (a) 1 M ZnSO_4_+1 M Na_2_SO_4_, (b) 1 M Na_2_SO_4_, and (c) 2 M ZnSO_4_ electrolyte.^117^ (d) Schematic illustration of Zn^2+^ and Na^+^ de/intercalation mechanism in HVO electrode. Cycling performance in 2 M ZnSO_4_ and 1 M ZnSO_4_ + 1 M Na_2_SO_4_ electrolytes at 1 A·g^–1^ of (e) HVO electrodes and (f) ZNVO electrodes. Reproduced with permission from ref 117. Copyright 2023 Elsevier.

As illustrated in Figure 11d, Na-ion intercalation into V_2_O_5_ occurs at higher potentials (∼0.8 V), followed by Zn-ion intercalation at lower potentials (∼0.55 V).

In contrast to the findings of Diem et al.,^37^ where cointercalation of Li and Mg ions into V_2_O_5_ was not observed in a Li–Mg hybrid battery, the results suggest that Na ions enable more favorable cointercalation than Li ions, likely due to differences in the diffusion/intercalation behavior. This was also reported by Hu et al. for VS_2_, where cointercalation was observed in Na–Mg hybrid batteries but not in Li–Mg hybrid batteries.^133^ Figures 11e and 10f further demonstrate the electrochemical performance of hydrated V_2_O_5_ (HVO) and Zn_0.3_Na_0.43_V_2_O_5_ (ZNVO).^117^ The addition of 1 M Na_2_SO_4_ in the electrolyte enhances the electrochemical stability compared to the electrolyte with solely Zn ions. At 1 A·g^–1^, the specific capacities of hydrated V_2_O_5_, Zn_0.3_Na_0.43_V_2_O_5_, and Na_0.33_V_2_O_5_ are 155, 192, and 131 mAh·g^–1^, respectively.

In summary, Na–Zn hybrid batteries have been investigated using various vanadium-based cathode materials, including NVP, NVPF, V_2_O_5_, Zn_0.3_Na_0.43_V_2_O_5_, and Na_0.33_V_2_O_5_, resulting in specific capacities ranging from 75 to 192 mAh·g^–1^ vs Zn/Zn^2+^. The observed Na-/Zn-ion intercalation into NVP closely resembles that of the Na /Mg ions, likely due to the similar ionic radii and charge of Mg and Zn ions.

The intercalation behavior depends more strongly on the anion species present in the electrolyte, such as the strong triflate anion (CF_3_SO_3_)^−^ or the weaker acetate anion (CH_3_COO)^−^, than on the Zn-ion concentration.

Hydrated V_2_O_5_, Zn_0.3_Na_0.43_V_2_O_5_, and Na_0.33_V_2_O_5_ show cointercalation of Zn and Na ions, which enhances electrochemical stability compared to systems based solely on Zn-ion intercalation in zinc-ion batteries. While earlier studies predominantly reported Zn^2+^/H^+^ cointercalation,^166,167^ Han et al. demonstrated through XRD, ICP-OES, and electrochemical analyses that a Zn-/Na-ion cointercalation occurs. However, it is credible that factors such as the Zn-ion concentration, electrolyte composition (including the type of anions in the electrolyte), and structural properties of the vanadium-based materials may lead to different intercalation behaviors for electrode materials with the same chemical formula.

In addition to the changes on the cathode, Gao et al. observed that the presence of Na ions in the electrolyte helps to suppress Zn dendrite formation on the Zn anode in a ZIB.^167^ These findings highlight the need for a deeper investigation of the anode as well as continued exploration of vanadium-based cathodes to enhance the understanding and optimization of Na–Zn hybrid battery performance.

### 5.4 Further Hybrid Batteries Based on Alkali and Multivalent Metal Cations

In this section, we highlight the potential of vanadium-based materials in various hybrid batteries. Although these materials are not widely used, they exhibit promising electrochemical properties that can be further investigated.

Several configurations utilizing vanadium-based cathode materials have recently been explored, including Li_3_V_2_(PO_4_)_3_ vs Zn/Zn^2+^ and V_2_O_5_ vs Zn/Zn^2+^ in Li–Zn hybrid batteries, NVP vs Zn/Zn^2+^ in Li–Na–Zn hybrid batteries, NVP vs Al/Al^3+^ in Na–Al hybrid batteries, and VS_2_ vs Mg/Mg^2+^ in K–Mg hybrid batteries. Consistent with previous findings on Li_3_V_2_(PO_4_)_3_ in Li–Mg hybrid batteries, Zhao et al. confirmed that the intercalation mechanism in these hybrid batteries follows a Daniel-type hybrid configuration.^104^ Specifically, Li ions undergo inter- and deintercalation within Li_3_V_2_(PO_4_)_3_ while reversible Zn plating takes place at the Zn metal anode.

When NVP is used instead of Li_3_V_2_(PO_4_)_3,_ both Li and Na ions cointercalate into the NVP structure, while Zn ions insertion/extraction remain restricted at the anode. This observation demonstrates the feasibility of hybrid batteries involving more than two ionic species. In terms of the electrochemical performance, Li_3_V_2_(PO_4_)_3_ exhibits a higher specific capacity of 128 mAh·g^–1^ and more redox peaks in the CV measurement, compared to NVP with 96 mAh·g^–1^ as shown in Figure 12a–d, which is primarily attributed to its higher theoretical capacity of 191 mAh·g^–1^ compared to 118 mAh·g^–1^ for NVP.^104^

**Figure 12:**
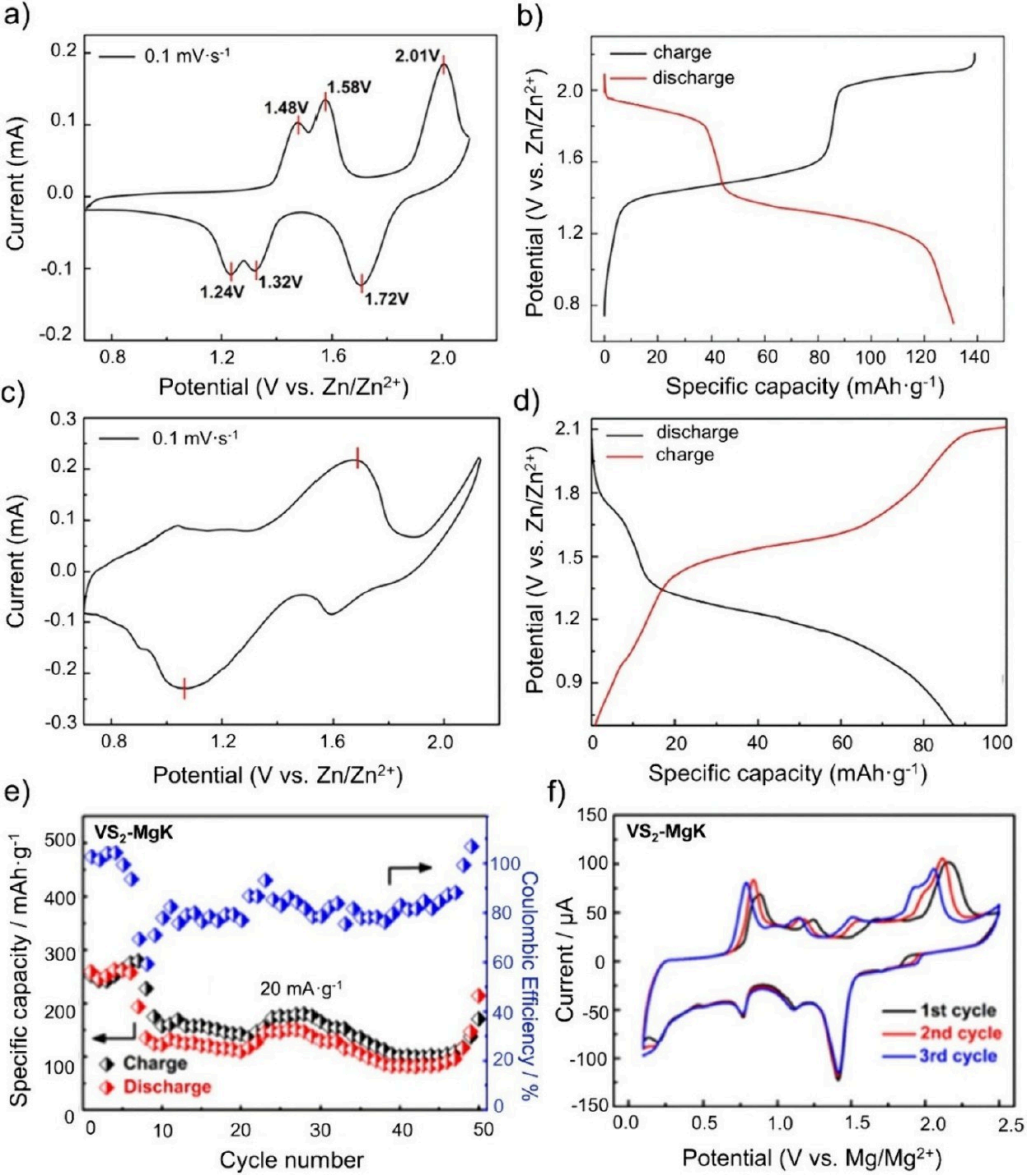
Electrochemical performance of (a, b) Li_3_V_2_(PO_4_)_3_ in a Li–Zn hybrid battery and (c, d) NVP in a Li–Na–Zn hybrid battery. Reproduced with permission from ref 104. Copyright 2016 Springer Nature. (e, f) VS_2_ in a K–Mg hybrid battery. Reproduced with permission from ref 133. Copyright 2021 ACS.

Recently, Reshma et al. reported V_2_O_5_ as cathode material in a Li–Zn hybrid battery using 0.5 M Zn­(ClO_4_)_2_ + 1 M LiClO_4_ as the electrolyte.^119^ They reached a specific capacity of 410.5 mAh·g^–1^ at a high current density of 5.7 A·g^–1^. Consistent with previous studies, they concluded that solely Li-ion inter- and deintercalation into V_2_O_5_ occurs while Zn-ion stripping/plating is occurring on the anode.

In another study, the electrochemical performance of NVP versus Al/Al^3+^ in a 2 M NaAlCl_4_/1-ethyl-3-methylimidazolium chloride (EMImCl) and aluminum chloride (1:1.1) electrolyte were analyzed.^115^ Aluminum offers several advantages over other electrode materials: it is highly abundant, has a small ionic radius, and possesses the highest volumetric specific capacity in mAh·cm^–3^ among the metals discussed in Table 1. De et al. showed that V_2_O_5_ as cathode material in an aluminum-ion battery (AIB) led to a specific capacity of ∼60 mAh·g^–1^ at 1 A·g^–1^ while exhibiting a capacity retention of 96% after 1000 cycles.^168^ In another study, Krabao et al. synthesized Al-doped Na_4‑x_VMn_1–*x*_Al_*x*_(PO_4_)_3_/C (0 ≤ *x* ≤ 0.5), which showed an improved electrochemical stability compared to unmodified Na_4_VMn­(PO_4_)_3_/C in SIBs.^169^

Although these studies show that Al/AIBs might be promising, they present notable challenges, including strong electrostatic interactions, sluggish kinetics, and side reactions with cathode materials, as seen with the APC electrolyte in MIBs.^170^ Despite these limitations, Sun et al. demonstrated reversible Al-ion inter- and deintercalation at the anode (Al metal), while Na ions can (partly) reversibly intercalate into the NVP cathode, leading to a specific capacity of 96 mAh·g^–1^.^115^ As some of the Na ions are trapped in the electrolyte, they further showed that increasing the salt concentration in the electrolyte reduces the Na ion trapping and leads to improved stability during charging and discharging of the cell.

In Figure 12e and f, Hu et al. reported a hybrid battery utilizing VS_2_ as the cathode, Mg metal as the anode, and an electrolyte composed of 3 mmol Mg­(HMDS)_2_ + 6 mmol AlCl_3_ in THF with 5 mmol KTFSI.^133^ This hybrid battery achieved a specific capacity of 250 mAh·g^–1^ at 0.05 A g^–1^, surpassing the capacities reported for Li–Mg and Na–Mg hybrid batteries by the same group. The enhanced performance is attributed to K ions, which expand the crystal structure and intercalation channels, consistent with prior findings that larger ions improve structural accessibility.^138,171^ However, K ions are approximately 1.35 times larger than Na ions and nearly twice the size (∼1.82 times) of Li ions, which also causes excessive volume expansion and results in irreversible structural damage, leading to a decrease of the stability during the electrochemical charge/discharge process, as observed in VS_2_ in the K–Mg hybrid battery.^133^ Reducing K-ion concentration may mitigate these effects by balancing structural integrity with improved ion transport.

In line with Gao et al. and^167^ Wan et al.^166^ that found that the addition of Na ions in a ZIB led rather to the suppression of the formation of Zn dendrites than to cointercalation of Na/Zn ions in the cathode, Pang et al. observed a similar trend using LiV_3_O_8_ nanorods vs a Zn/Zn^2+^ in a 3 M Zn­(CF_3_SO_3_)_2_ + 0.5 M Li­(CF_3_SO_3_) aqueous electrolyte.^172^ Ex situ XPS analysis confirmed that only Zn ions intercalate into the cathode, while Li ions inhibit the formation of the Zn_3_(OH)_2_V_2_O_7_·2H_2_O byproduct and dendrite growth, leading to enhanced stability. This observation contrasts with previous studies on Li–Zn hybrid batteries and may be attributed to several factors, as discussed in the previous sections.

## 6 Vanadium-Based Hybrid Batteries Combined with Membranes or Hybrid Electrolytes

Hybrid electrolytes (quasi-solid-state electrolytes (QSSE)) have gained attention due to their inherent safety as opposed to standard commercial liquid electrolytes and their potential compatibility with a Li-metal anode.^173–175^ Liquid electrolytes include organic solvents that are flammable, which are bypassed when using QSSEs such as polymers.

Kravchyk et al. proposed combining a Na_1.5_VPO_4.8_F_0.7_ cathode and magnesium anode in a cell with a liquid electrolyte and a sodium-ion conducting β-alumina membrane as shown in Figure 13a.^30^ Their membrane allowed for Na ions to freely move from the sodium liquid electrolyte to the Na/Mg liquid electrolyte, resulting in Mg/Na_1.5_VPO_4.8_F_0.7_ cells that performed with a high average discharge voltage of 3.0 V and a specific capacity of 110 mAh·g^–1^. Due to the large electrolyte mass needed to supply all ions necessary for operation and the reversibility dependence of magnesium plating on the concentration of Mg ions, the solvability of magnesium ions was limiting the factor for energy density calculations. Despite this, Kravchyk et al. showed an energy efficiency of 90% with an energy density of 57 Wh·kg^–1^, which is comparable to that of vanadium redox-flow batteries.^30^

**Figure 13:**
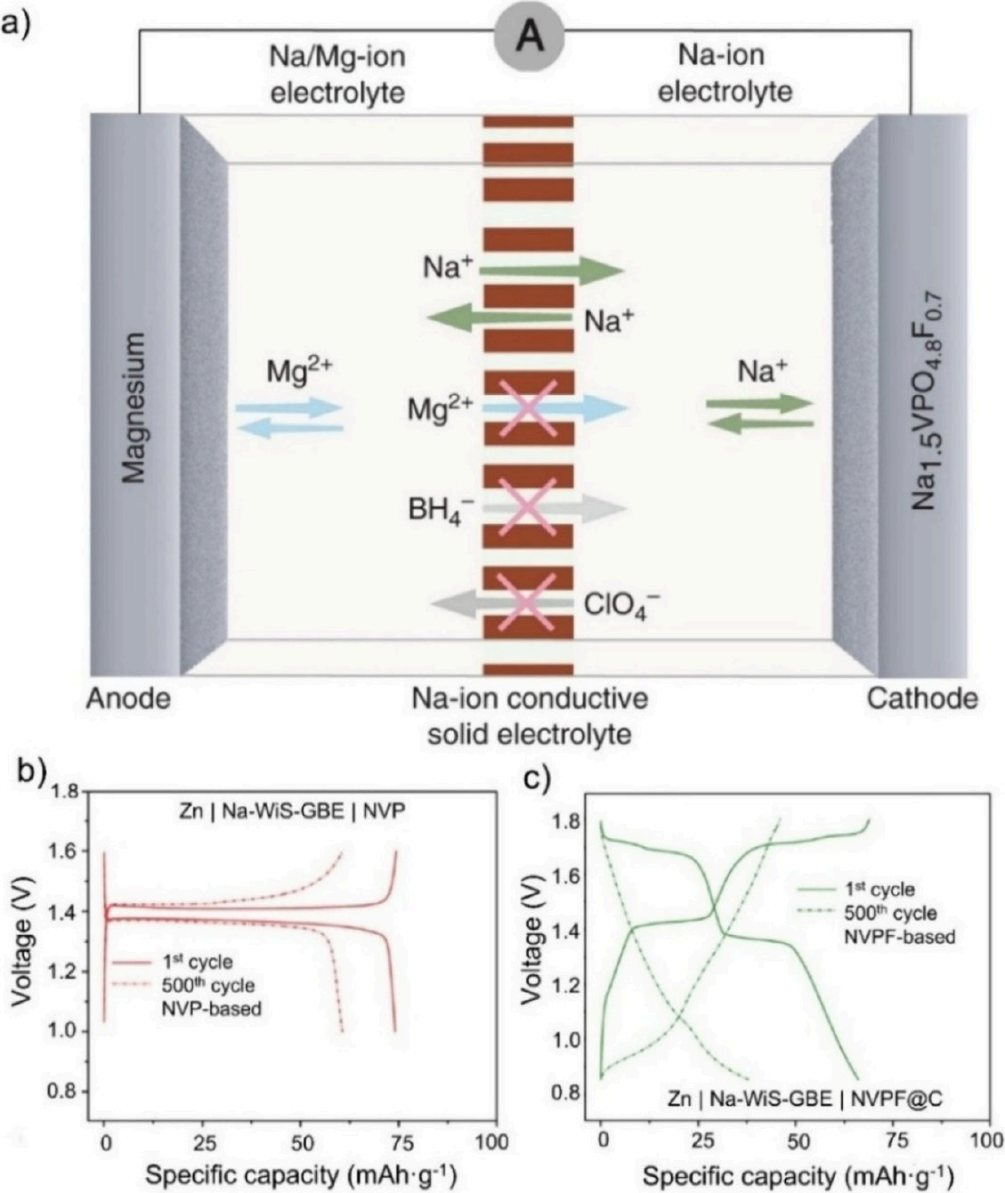
(a) Working principle and schematic of the charge/discharge processes of a high-voltage Mg/Na dual-ion battery. Reproduced with permission from ref 30. Copyright 2019 Springer Nature. (b, c) Initial and final GCD profiles of WiS-GBEs with NVP and NVPF as cathode material. Reproduced with permission from ref 114. Copyright 2024 ACS.

Kasprzak et al. demonstrated how a QSSE, in this case a water-in-salt gel biopolymer, could be used to combine Zn and Na ions.^114^ The water-in-salt gel biopolymer is a compromise between fully solid electrolytes and liquid electrolytes, with the Zn/Na bisalt encapsulated by a cellulose membrane. This membrane is flexible but also highly conductive and shows a wide electrochemical stability window. In addition to their investigation on a NaZnHCF cathode, they showed that the QSSE can be combined with NVP or NVPF in a Na–Zn hybrid battery leading to initial specific capacities of 65–75 mAh·g^–1^ (see Figure 13b and c).

Vanadium-based materials in metal–ion hybrid batteries with membranes or QSSEs remain largely unexplored, with many research opportunities to contribute to the understanding of QSSEs in hybrid batteries. It remains unclear to what extent the electrochemical properties observed with conventional liquid electrolytes can be adapted to hybrid electrolytes. Parameters such as low ionic conductivity and increased interfacial resistance may lead to performance limitations, making the evaluation of hybrid batteries even more challenging.^176^

Nevertheless, higher operational safety and the possibility to adjust cells with wide potential ranges compared with liquid electrolytes underscore the importance of investigating QSSEs in hybrid batteries, not only to optimize vanadium-based electrodes in hybrid batteries but also to advance the broader development of next-generation energy-storage batteries.

## 7 Challenges and Perspectives

Despite considerable achievements in the use of vanadium-based materials in hybrid batteries, several fundamental mechanisms remain insufficiently understood and need to be further explored.

### 7.1 Unresolved Intercalation Mechanism

It is often discussed in the respective publications and verified by appropriate characterization methods, such as operando XRD, which intercalation behavior (Daniel type, cointercalation type, or rocking chair type) was observed in vanadium-based materials. The most extensively investigated vanadium-based cathode material is Na_3_V_2_(PO_4_)_3_ (NVP) and its modifications (such as NVPF) in Li–Na hybrid batteries. They show comparable electrochemical performances and intercalation behavior regardless of the anode used such as Li metal, Li_4_Ti_5_O_12_, or carbon-based materials using LiPF_6_ or LiClO_4_ dissolved in EC, DMC, and/or DEC as electrolyte. It is mainly reported that during the initial charging process, two Na ions are extracted from NVP via a solid-solution process, resulting in the transformation of Na_3_V_2_(PO_4_)_3_ into NaV_2_(PO_4_)_3_. During the following discharge cycle, Li ions are inserted into the structure, leading to the phase formation from NaV_2_(PO_4_)_3_ to Na_3‑x_Li_*x*_V_2_(PO_4_)_2_. This behavior is commonly attributed to the smaller ionic radius and lower atomic mass of Li compared to Na.

In the case of Na_3_V_2_(PO_4_)_2_F_3_ (NVPF), it is typically reported in the literature to crystallize in the tetragonal structure (space group *P*4_2_ */mnm*). However, if a subtle orthorhombic distortion is present in NVPF, it might be synthesized in the space group Amam.^177^ If this is the case, changes in the space group from Na_3_V_2_(PO_4_)_2_F_3_ (*Amam*) to Na_2‑x_Li_*x*_V_2_(PO_4_)_2_F_3_ (*I*4*/mmm*) and further Na_1–*x*_Li_*x*_V_2_(PO_4_)_2_F_3_ (*Cmc*2_1_) are observed, during the galvanostatic charge process, which are reversible during the discharge process.^95^ However, a reliable detection of such an orthorhombic distortion in NVPF, and thus a verification if NVPF was synthesized in the space group *P*4_2_ */mnm* or *Amam*, requires high angular resolution synchrotron radiation diffraction, which has normally not been fully investigated in the reported studies.

Furthermore, if NVP is used as the cathode material in hybrid batteries other than Li–Na, different observations and mechanisms have been reported. In two studies using NVP as cathode material in Na–Mg hybrid batteries, NVP transformed once into Mg_0.4_Na_2.1_V_2_(PO_4_)_3_ and in the other case to Mg_0.79_Na_1.02_V_2_(PO_4_)_3_ after a complete galvanostatic cycle, which show a different behavior in the same Na–Mg hybrid battery system. In a Na–Zn hybrid battery, a quasi-two-stage intercalation process was observed, and two phases, namely, NVP and Zn_0.25_NaV_2_(PO_4_)_3_, were identified after a full galvanostatic cycle.

These results indicate that the intercalation behavior of NVP in Na–Mg and Na–Zn hybrid batteries differs from that in Li–Na hybrid batteries. This may be attributed to the higher polarization effect generated by the divalent charges or the higher migration barrier of multivalent ions compared to Li-ions.

In general, the intercalation mechanism in hybrid batteries depends on numerous factors, which are listed here and might still not be complete:

- The combination of the incorporated ions
- The concentration of the ions inside the battery and their ratio to each other
- The energy needed to overcome the migration barrier of these ions into the structure of the electrode material
- The energy difference required for overcoming the migration barrier of the used ions
- The anions in the electrolyte and how strong they interact with the incorporated ions
- The solution in the electrolyte
- The anode material (counter electrode)
- The interlayer distance of the cathode material
- Modifications, such as composite formation etc., on the cathode material
- Polarization/conductivity effects of the used cathode material, which may lead to side reactions during the charge/discharge process
- The current density and the potential window

Consequently, further in-depth investigations, particularly focusing on the electrodes and electrolyte, are required to achieve a more comprehensive understanding of the influence of the above-mentioned factors. These investigations should be carried out for NVP and its modifications in Li–Na hybrid batteries, as this system is currently the most well understood. Afterward, it can serve as a role model, for both experimental (e.g., operando/synchrotron XRD) and modeling methods (e.g., density functional theory (DFT)), in other hybrid battery types.

### 7.2 Role of the Second Introduced Ion in the Electrolyte and on the Anode

Although intercalation in hybrid batteries is typically described using Daniel type or cointercalation mechanisms, the second introduced ion may still interact, e.g., with the counter electrode, which influences electrochemical behavior. Previous publications have demonstrated that the addition of Li or Na ions in zinc-ion batteries (ZIBs) can suppress dendrite formation on the Zn anode and hamper the formation of undesired Zn_3_(OH)_2_V_2_O_7_·2H_2_O byproducts. In this context, it would be interesting to investigate whether the incorporation of Li or Na ions leads to similar effects in other hybrid battery types (e.g., on Na metal in SIBs) or if other factors, such as the aqueous-based electrolytes commonly used in ZIBs, are crucial for this observation.

### 7.3 Irreversible Structural Changes Induced by Na- or K-Ion Cointercalation

The incorporation of larger Na and K ions in LIBs, ZIBs, or MIBs leads often to the expansion of the crystal structure and the intercalation channels within the cathode materials. However, this expansion has its limits. Analyzing the structural changes of VS_2_ as a cathode material in a Mg–K hybrid battery has shown that the inter- and deintercalation of K ions was not reversible due to excessive volume changes that caused irreversible structural damage.

In regard to the long-term stability of these cathode materials, it is necessary to determine to which concentration or degree of incorporation of large ions the associated structural expansion remains stable. Key questions include the ion concentration at which structural changes become detectable. At this point, it needs to be evaluated whether changes in the electrochemical performance are already observable at this stage or if a “lag” exists between structural and electrochemical responses. In addition, it remains unclear whether, and to what extent, Na- and K-ion cointercalation is beneficial or affects the electrochemical performance of cathode materials with an already expanded interlayer space.

## 8 Conclusions

This review summarizes the intercalation behavior and electrochemical performance of vanadium-based materials as cathode materials in metal–ion hybrid batteries. Figure 14 provides an overview of these vanadium-based materials ranked by their reported specific capacities. Among those, Na_3_V_2_(PO_4_)_3_ is the most extensively studied in Na-containing hybrid batteries as two Na ions can reversibly be inter- and deintercalated during galvanostatic cycling. Consequently, notable specific capacities have been achieved, including 148 mAh·g^–1^ in Li–Na hybrid cells at 0.1 C, and 96 mAh·g^–1^ in both Na–Al and Li–Na–Zn hybrid batteries at 0.2 C. Li-x-based hybrid batteries in combination with Mg or Zn show the highest specific capacities reported, exemplified by V_2_O_5_ (410 mAh·g^–1^ at 5.7 A·g^–1^) and NaV_3_O_8_·1.69H_2_O (0.02 A·g^–1^).

**Figure 14:**
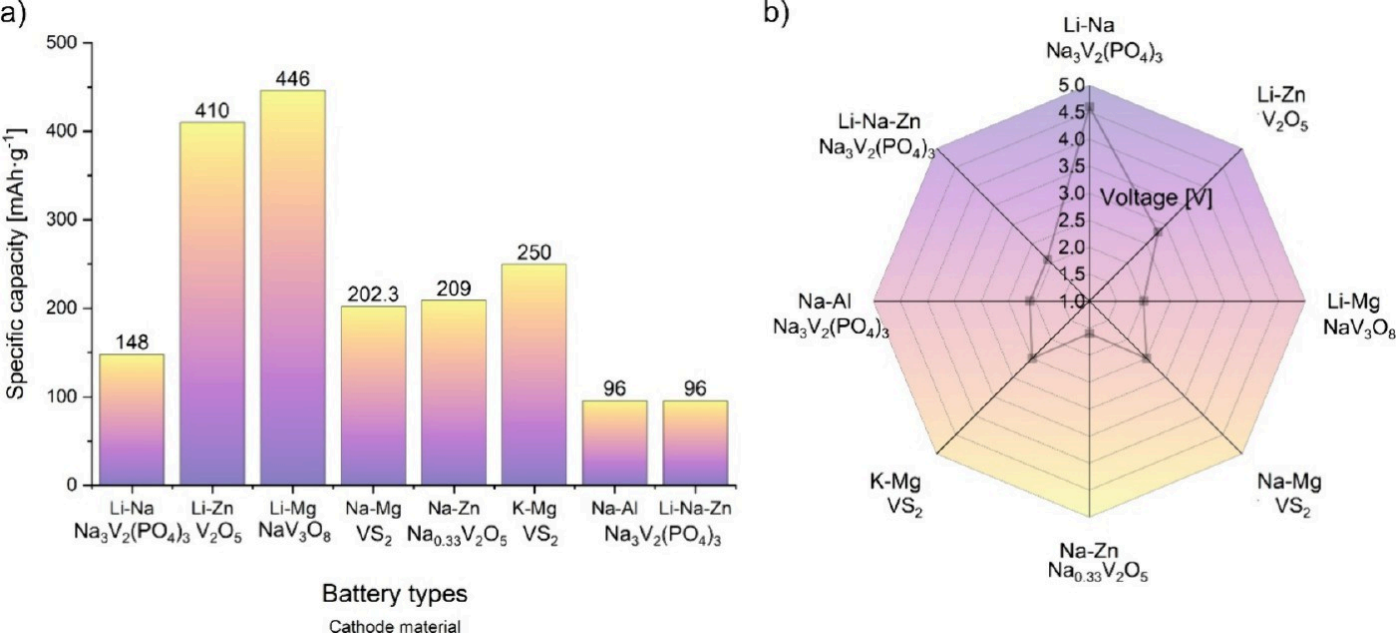
Overview of the electrochemical performance of vanadium-based materials in metal–ion hybrid batteries. (a) Overview of the specific capacities of Na_3_V_2_(PO_4_)_3_ vs Li/Li^+^ in Li–Na (0.1C),^93^ Na_3_V_2_(PO_4_)_3_ vs Al/Al^3+^ in Na–Al (0.2C),^115^ and Na_3_V_2_(PO_4_)_3_ vs Zn/Zn^2+^ in Li–Na–Zn (0.2C),^104^ V_2_O_5_ vs Zn/Zn^2+^ in Li–Zn (5.7 A·g^–1^),^119^ NaV_3_O_8_·1.69H_2_O vs Mg/Mg^2+^ in Li–Mg (0.02 A·g^–1^),^123^ Na_0.33_V_2_O_5_ vs Zn/Zn^2+^ in Na–Zn (1 A·g^–1^),^117^ and VS_2_ vs Mg/Mg^2+^ in Na–Mg (0.05 A·g^–1^),^133^ and K–Mg (0.05 A·g^–1^)^133^ hybrid batteries. (b) Comparison of the highest applied voltage for the vanadium-based materials illustrated in (a) across various hybrid batteries.

In combination with Mg metal as the counter electrode, VS_2_ delivers capacities of 202.3 mAh·g^–1^ in Na–Mg and 250 mAh·g^–1^ in K–Mg hybrid batteries at 0.05 A·g^–1^. Figure 14b illustrates the highest applied potential used in the potential windows during the studies in Figure 14a. In general, the applied voltage depends on the electrode materials used as well as the electrolyte. In this context, the Li–Na hybrid battery reached the highest operating voltage of 4.6 V vs Li/Li^+^, which is consistent with the low standard potentials of Li (−3.05 V vs SHE) and Na (−2.71 V vs SHE). Although other combinations such as Na and Mg (−2.36 V vs SHE) show also low redox potential combinations, the achievable voltages are limited by the stability of the used complex all-phenyl electrolyte, which is necessary in the combination with the use of Mg metal as the counter electrode. In aqueous electrolytes, which are often used in Na–Zn hybrid batteries, the maximum potential is further constrained to <2.0 V vs Zn/Zn^2+^ to prevent side reactions (i.e., hydrogen evolution).

Table 3 summarizes the performance of vanadium-based materials in single-ion batteries. The results indicate that the achieved specific capacities are often comparable to those reported in hybrid batteries (see Table 2). In the case of V_2_MoO_8_, V_3_O_7_·H_2_O and VS_4_ in MIBs, Li–Mg hybrid batteries exhibit an improved electrochemical performance and demonstrate that they can broaden the range of viable cathode materials, even for more complex configurations, such as APC electrolytes in combination with Mg-metal.

**Table 3:** Electrochemical Performance of Several Vanadium-Based Materials in LIBs, SIBs, MIBs, ZIBs, AIBs, and KIBs

Vanadium-based material and counter electrode	Electrolyte	Hybrid battery ion type	Specific capacity [mAh·g^–1^]/C-rate or current density [A·g^–1^]	Poten-tial window [V]	Ref
Li_3_V_2_(PO_4_)_3_/C vs Li/Li^+^ (Li metal)	1 M LiPF_6_ in EC:EMC:DMC (1:1:1 vol %)	LIB	122.2/0.2 C	3.00–4.30	^178^
Na_3_V_1.9_Ti_0.1_(PO_4_)_3_/C vs Na/Na^+^ (Na metal)	1 M NaClO_4_ in EC:PC (1:1 vol %) with 5% FEC	SIB	123.3/0.1 C	2.00–4.20	^179^
Nitrogen-doped carbon layer-coated Na_3_V_2_(PO_4_)_3_ composite material vs Na/Na^+^ (Na metal)	1 M NaClO_4_ in EC:PC (1:1 vol %) with 5% FEC	SIB	109.18/1 C	2.30–4.20	^180^
V_2_O_5_/MCMB vs Na/Na^+^	1 M NaClO_4_ in EC:PC (1:1 vol %) with 5% FEC	SIB	99.65/0.5	1.50- 3.80	^66^
V_3_O_7_·H_2_O vs Mg/Mg^2+^ (Mg metal)	0.25 M APC	MIB	0/0.025	0.50–2.00	^36^
V_2_MoO_8_ vs Mg/Mg^2+^ (Mg metal)	0.4 M APC	MIB	200 (initial discharge capacity)/0.02	0.50–2.40	^127^
			The discharge capacity for the second cycle is ∼60 mAh·g^–1^		
VS_4_ nanodendrites vs Mg/Mg^2+^ (Mg metal)	0.4 M APC	MIB	∼190/0.5	0.20–2.20	^81^
			Initial cycle (152 mAh·g^–1^ (discharge) and 85 mAh·g^–1^ (charge))		
Zn_0.25_V_2_O_5_·H_2_O vs Zn/Zn^2+^ (Zn metal)	1 M ZnSO_4_ in H_2_O	ZIB	275/1C	0.50–1.40	^181^
Layered VS_2_ vs Zn/Zn^2+^ (Zn metal)	1 M ZnSO_4_ in H_2_O	ZIB	190/0.05	0.40–1.00	^182^
Binder-free and self-supporting Cu/V_2_O_5_ vs Al/Al^3+^ (Al foil)	1-ethyl-3 methylimidazolium chloride mixed with aluminum chloride in the ratio of 1:1.5	AIB	∼170/0.025	0.20–1.10	^15^
V_3_O_7_·H_2_O vs AC	0.5 M KPF_6_ in EC/DEC (1:1 vol %)	KI-Cell	168/0.005	–1.60–0.80	^183^
K_2_(VO)_3_(P_2_O_7_)_2_ vs K/K^+^ (K metal)	9 M potassium bis(fluorosulfonyl)imide (KFSI) in EC/DEC (1:1 vol %)	KIB	82/0.01	1.70–4.80	^184^

Besides the electrochemical performance, the structural and morphological behavior of the vanadium-based materials have further been analyzed using a variety of characterization methods such as (operando) X-ray diffraction (XRD), X-ray photoelectron spectroscopy (XPS), X-ray absorption spectroscopy (XAS), extended X-ray absorption fine structure (EXAFS), Fourier-transform infrared spectroscopy (FTIR), Raman spectroscopy (Raman), scanning electron microscopy (SEM), high-resolution transmission electron microscopy (HRTEM), atomic force microscopy (AFM), selected area electron diffraction (SAED), energy dispersive X-ray spectra (EDS), and density functional theory (DFT). The results indicate that the probability of cointercalation in vanadium-based cathode materials is generally higher in Na-containing hybrid batteries than in Li-containing hybrid batteries. This difference may arise from the faster diffusion of Li ions, due to its small size and mass.

Li–Mg hybrid batteries were investigated in numerous cathode materials, showing an intercalation mechanism similar to a Daniel type (e.g., for V_2_O_5_, V_3_O_7_·H_2_O or NaV_3_O_8_·1.69H_2_O) as well as a cointercalation type (e.g., for Li_0.15_V_2_C and VS_4_).

The lower tendency of Mg ions to cointercalate in oxide-based vanadium compounds is likely attributable to its higher migration energy barriers compared to, for example, sulfides. In Na–Mg hybrid batteries, in addition to the expanded structure by Na-ion intercalation, these migration energy barriers may be closer to each other, which may further explain the more frequent observation of a cointercalation mechanism for the same electrode materials compared to Li–Mg hybrid batteries. Na–Zn hybrid batteries generally follow a trend similar to that of Na–Mg hybrid batteries. Other hybrid batteries, such as Mg–K or Na–Al batteries and incorporating quasi-solid-state electrolytes, have been explored only preliminarily for vanadium-based cathodes. Initial results suggest that they are compatible with hybrid-ion batteries, but their capabilities remain largely unexplored and need to be further analyzed in future studies.
